# Simulation of nitrogen nuclear spin magnetization of liquid solved nitroxides†

**DOI:** 10.1039/d0cp06071b

**Published:** 2021-07-29

**Authors:** Andriy Marko, Antonin Sojka, Oleksii Laguta, Petr Neugebauer

**Affiliations:** Central European Institute of Technology, Brno University of Technology Purkynova-Str. 123 61200 Brno Czech Republic amarko@ceitec.vutbr.cz

## Abstract

Nitroxide radicals are widely used in electron paramagnetic resonance (EPR) applications. Nitroxides are stable organic radicals containing the N–O˙ group with hyperfine coupled unpaired electron and nitrogen nuclear spins. In the past, much attention was devoted to studying nitroxide EPR spectra and electron spin magnetization evolution under various experimental conditions. However, the dynamics of nitrogen nuclear spin has not been investigated in detail so far. In this work, we performed quantitative prediction and simulation of nitrogen nuclear spin magnetization evolution in several magnetic resonance experiments. Our research was focused on fast rotating nitroxide radicals in liquid solutions. We used a general approach allowing us to compute electron and nitrogen nuclear spin magnetization from the same time-dependent spin density matrix obtained by solving the Liouville/von Neumann equation. We investigated the nitrogen nuclear spin dynamics subjected to various radiofrequency magnetic fields. Furthermore, we predicted a large dynamic nuclear polarization of nitrogen upon nitroxide irradiation with microwaves and analyzed its effect on the nitroxide EPR saturation factor.

## Abstract 2

Simulated nitrogen nuclear spin magnetization generated by RF and MW irradiation of liquid solved nitroxides.

## Graphical abstract

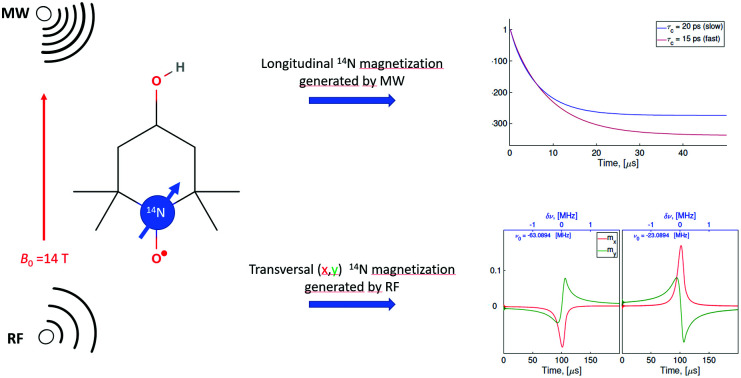

## I. Introduction

**Fig. 1:**
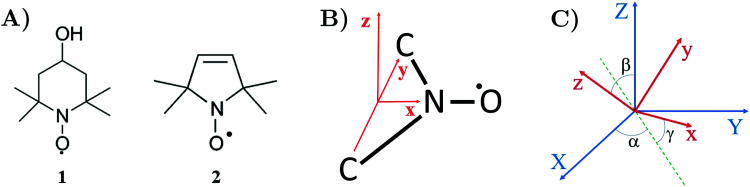
(A) Examples of nitroxide radicals: **1** – (4-hydroxy-2,2,6,6-tetramethylpiperidin-1-yl)oxyl commonly known as TEMPOL. **2** – (2,2,5,5-tetramethylpyrrolidine-1-yl)oxyl. (B) The fragment of nitroxide radical containing a nitrogen atom N and three atoms O, C, C covalently bound to N. {***x***,***y***,***z***} is the coordinate frame associated with nitroxide. Its ***x***-axis connects atoms N and O and ***y***-axis links the two carbon atoms bound to N. (C) Orientation of the nitroxide molecular frame {***x***,***y***,***z***} in the laboratory coordinate system {***X***,***Y***,***Z***} determined by the Euler angles (*α*,*β*,*γ*).

Organic nitroxide radicals play a vital role in EPR spectroscopy.^1,2^ Nitroxides are stable molecules with an unpaired electron (see Fig. 1A), which is usually localized in the middle of the N–O˙ bond, featuring a strong hyperfine interaction with the nitrogen nuclear spin.^3^ Various materials, which are usually EPR silent, can be made suitable for EPR experiments by introducing nitroxides into investigated samples. By shape analysis of nitroxide EPR spectra, one can monitor such parameters as viscosity, polarity of micro-environment, proticity and the presence of oxygen.^4–7^ Also, nitroxides can be used as a polarizing agent for Dynamic Nuclear Polarization (DNP) to enhance Nuclear Magnetic Resonance (NMR) signals.^8–10^ Since nitroxides can be attached to large macromolecules, they are often employed as spin labels to access valuable information about the structure and dynamics of organic polymers and bio-molecular systems. In frozen solutions, spin-labeled molecules can be effectively studied by the pulsed EPR technique at low temperatures.^11^ For example, nitroxide spin labels combined with pulsed electron–electron double resonance can detect macromolecule conformational flexibility at the nanometer scale.^12–16^ Recent methodological developments aim to extend the sensitivity of this technique to the range above 10 nm.^17–19^

EPR spectra are sensitive to the rotational motion of radicals in liquids. Description of the shape of EPR spectra is determined by the time scale of random rotations, which cause fluctuations of magnetic spin interactions. For a fast radical motion, when the time scale of random fluctuations is much shorter than the changes in the spin system density matrix, EPR spectra can be described in the frames of Bloch–Wangsness–Redfield density matrix perturbation theory, which is frequently used in NMR.^20–24^ Usage of viscous solvents or attachment of nitroxides as spin labels to large macromolecules in liquid solutions can strongly affect their dynamical characteristics, which do not allow anymore to treat nitroxides molecular dynamics in the fast motion regime or to consider them as the isotropic Brownian rotational diffusion. To describe slow motion EPR spectra, several theoretical approaches have been developed.^25^ Quite some time ago, linear response theory involving perturbation theory was employed to analyze EPR spectra of the system beyond the fast motion limit.^26–28^ Computation of EPR spectra *via* the solution of the stochastic Liouville equation (SLE) with the nitroxide spin label motion represented by diffusion operator was very successful.^29,30^ The software packages based on this method played a very important role in many structural and dynamics studies of large macromolecules.^31–34^ To account for complex spin label dynamics affected by macromolecular environment such as spin labeled proteins in solution, the methods to obtain EPR spectra from molecular dynamics trajectories are developed.^35–40^

Despite the fact that the EPR response of the nitroxide unpaired electron spin has been studied under various conditions experimentally as well as theoretically, the nitroxide nitrogen nuclear spin dynamics has not been investigated in detail so far. The major difficulty in observing the nitrogen nuclear spin magnetization is caused by the strong hyperfine coupling, which leads to short nuclear spin relaxation times and large broadening of NMR lines. In this work, we attempted to predict the nitroxide nitrogen nuclear spin dynamics in various magnetic resonance experiments on liquid samples. To this end, we employed a nitroxide spin Hamiltonian including typical electron and nuclear spin interactions, which are modulated by a fast stochastic motion of the molecular isotropic rotational diffusion process. We computed the nuclear spin magnetization by solving numerically the master Liouville/von Neumann (LvN) equation for the spin system density matrix in semi-classical approximation.^24^ The solutions were computed using the spin relaxation operator obtained by the Monte-Carlo method to model stochastically changing magnetic spin interactions and ensemble averaging. Here, it should be mentioned, that the computational approach, which we adapted in our work, is not the only way to deal with nitroxide spin dynamics. Also, other existing methods based on Bloch–Wangsness–Redfield perturbation theory, solution of stochastic Liouville equation or usage of molecular dynamics trajectories, could be employed for the computation of nitroxide spin magnetization evolution.^25,40,41^ The simulation results, especially those which we obtained for the nitroxide EPR spectra, could be obtained using the functions built in the widely used spin dynamics software suites such as, *e.g.*, EasySpin or Spinach.^33,34^ We have chosen our computational approach based on a direct solution of the LvN equation since it has a straight forward concept, can be relatively easy repeated starting from scratch, and allows us to clearly follow all details of nitroxide nitrogen nuclear spin dynamics. The numerical algorithm developed in this work enabled us to simulate experiments with complicated time-dependence of magnetic fields *B*_0_ and *B*_1_ including the variation of magnitude, frequency, and phase.

Furthermore, special attention was devoted to analyzing the nitrogen nuclear spin dynamics upon the application of microwave irradiation with an electron spin resonance frequency. We predicted a large DNP of nitrogen by irradiation of nitroxide EPR line and investigated the effect of nitrogen nuclear spin dynamics on the nitroxide EPR saturation factor. This analysis might help to understand all DNP transfer mechanisms, which are being currently examined intensively.^42–45^ The computational concept elaborated in this work can also be used to simulate frequency scan EPR experiments.^46^

## II. Theory and methods

### A. Nitroxide spin Hamiltonian

To define nitroxide magnetic interaction tensors, a molecular frame with the *x*-axis along the NO bond is introduced, as shown in Fig. 1B. In this research, we focus on liquid solved nitroxides, which can randomly rotate in the solvent due to thermal motion. This rotation leads to the reorientation of the nitroxide molecular frames and, consequently, to the variation of the magnetic interaction tensors in the laboratory coordinate system linked to the spectrometer static magnetic field **B**_0_. The rotating nitroxide spin system is described by a time-dependent Hamiltonian, 
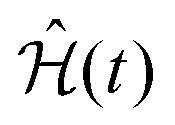
, which is expressed *via* the time-dependent magnetic interaction tensor values in the laboratory coordinate system and, of course, *via* the electron and nuclear spin operators **Ŝ** and **Î**, respectively. To simplify our derivations, we will further use the Hamiltonian operator divided by the Planck constant *ħ*, *i.e.*, 
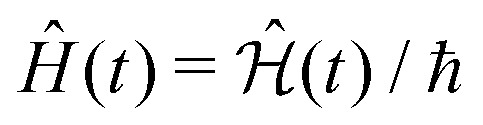
. Generally, we assume that *Ĥ*(*t*) consists of (i) electron and nitrogen nuclear Zeeman interaction terms *Ĥ*^e^_Z_(*t*) and *Ĥ*^N^_Z_, respectively, (ii) nitrogen nuclear quadrupole interaction *Ĥ*^N^_Q_(*t*), (iii) electron nitrogen nuclear hyperfine coupling *Ĥ*_HF_(*t*), and (iv) coupling of the electron spin to the radical rotational motion *Ĥ*_SR_(*t*). Hence, *Ĥ*(*t*) is presented as

1 *Ĥ*(*t*) = *Ĥ*^e^_Z_(*t*) + *Ĥ*^N^_Z_ + *Ĥ*^N^_Q_(*t*) + *Ĥ*_SR_(*t*) + *Ĥ*_HF_(*t*).

The electron Zeeman interaction Hamiltonian term is explicitly given by the following expression:2
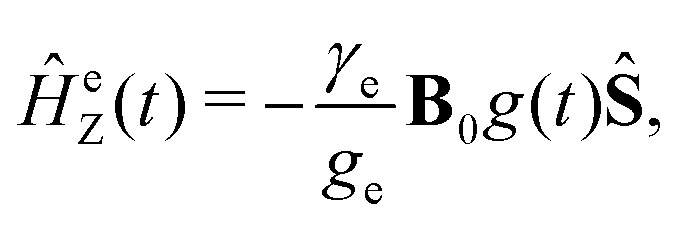
wherein *γ*_e_ ≈ −1.76086 × 10^11^ rad (T s)^−1^ is the electron magnetogyric ratio, *g*_e_ ≈ 2.0023 is the *g*-factor of free electron and *g*(*t*) is the value of the time-dependent nitroxide *g*-tensor in the laboratory coordinate system. In the principal axes, which in a good approximation coincide with the molecular frame defined in Fig. 1B, the value of *g*-tensor is constant. For our illustrative simulation, we will choose the typical principal axis *g*-tensor value *g*^pa^ = diag(2.0088, 2.0066, 2.0022). For the fitting of the real experimental EPR spectra discussed later (shown in the ESI†), we will use a slightly different *g*^pa^ value, which can depend on the solvent type, temperature, specification of nitroxide molecule, *etc.*

A similar formula to (2) is used to express the nitrogen nuclear spin Zeeman interaction,

3 *Ĥ*^N^_Z_ = −*γ*_N_**B**_0_**Î**,

with the nitrogen magnetogyric ratio *γ*_N_ ≈ 1.9331 × 10^7^ rad (T s)^−1^ for the isotope ^14^N and *γ*_N_ ≈ −2.7116 × 10^7^ rad (T s)^−1^ for the isotope ^15^N. In comparison to the electron Zeeman interaction, the nuclear Zeeman interaction anisotropy is neglected and *H*^N^_Z_ is assumed to be time independent.

The nitroxide hyperfine coupling between the unpaired electron and nitrogen nuclear spins is described by

4 *Ĥ*_HF_(*t*) = **Î***A*(*t*)**Ŝ**,

wherein *A*(*t*) is the time-dependent hyperfine interaction tensor in the laboratory coordinate system. In this work, we use the hyperfine tensor principal axis value equal to *A*^pa^ = 2π × diag(15, 15, 90) × 10^6^ rad s^−1^.

In the case of the isotope ^14^N with a spin 1, the nitroxide nitrogen nucleus exhibits quadrupole interaction described by the Hamiltonian,

5 *Ĥ*^N^_Q_(*t*) = **Î***Q*(*t*)**Î**,

wherein *Q*(*t*) is the quadrupole interaction tensor which has the principal axis value equal to *Q*^pa^ = 2π × diag(0.1, 1.6, −1.7) × 10^6^ rad s^−1^.^47,48^

In order to correctly predict relaxation times of electron spin magnetization in EPR experiments, the coupling of electron spin to nitroxide rotation was introduced.^49–51^ It is given by the Hamiltonian term

6 *Ĥ*_SR_(*t*) = −***η***(*t*)(*g*(*t*) − *g*_e_1_3_)**Ŝ**,

where ***η***(*t*) is the angular velocity of the nitroxide moiety.

Here we consider samples with a low nitroxide concentration neglecting Heisenberg spin exchange interaction, which can change EPR spectra significantly.^52–54^ Also, we neglect the translational diffusion of nitroxides, which can cause additional spin relaxation in inhomogeneous magnetic fields.^24,55,56^ For the simplification of our analysis, we assume that the principal axes of all magnetic tensors coincide, although the magnetic tensors with arbitrary principal axes orientations can be treated with almost the same efforts in our computation approach.

In order to determine the time-dependent laboratory frame magnetic tensors, which enter (2), (4), (5) and (6), from their principal axes values we will use the formula,

7 *X*(*t*) = *T*^−1^(**Ω**(*t*))*X*^pa^*T*(**Ω**(*t*)),

where *X* can be *g*, *A* or *Q* tensor. and *T*(*t*) is the time-dependent transformation matrix of the molecular frame to the laboratory coordinate system determined by the Euler angles **Ω**(*t*) = (*α*(*t*),*β*(*t*),*γ*(*t*)), (see Fig. 1C).

### B. The master equation

For the description of state evolution of nitroxide spin systems we employ a standard quantum statistical method in which the state of an ensemble of identical spin systems at a time point *t* is represented by the density matrix, *σ̂*(*t*). The time evolution of the density matrix is determined using the LvN equation,8
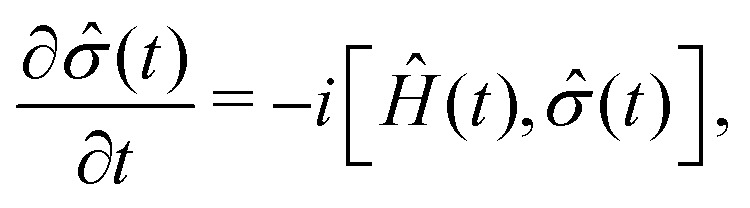
where *Ĥ*(*t*) is the spin system Hamiltonian described in the previous section. For liquid solved radicals the time-dependent Hamiltonian *Ĥ*(*t*) is assumed to consist of a stationary part *Ĥ*_0_ and a stochastically changing part *Ĥ*_1_(*t*), *i.e.*,^24,57^

9 *Ĥ*(*t*) = *Ĥ*_0_ + *Ĥ*_1_(*t*).

In our case *Ĥ*_0_ is the isotropic parts of Zeeman and hyperfine interactions given by10
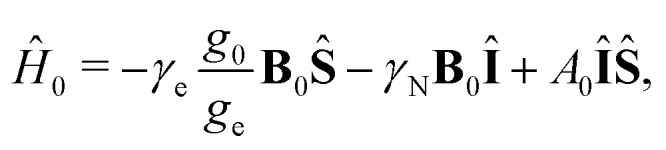
wherein *g*_0_ and *A*_0_ are the averaged values of the *g*- and hyperfine interaction tensors, *g*_0_ = trace{*g*}/3 and *A*_0_ = trace{*A*}/3, respectively. For the assumptions made later we present *Ĥ*_1_(*t*) as a sum of the two terms

11 *Ĥ*_1_(*t*) = *Ĥ*_1a_(*t*) + *Ĥ*_SR_(*t*),

wherein12
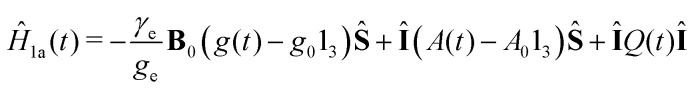
includes anisotropic parts of Zeeman, hyperfine and quadrupole interactions. In order to determine the density matrix for our system we follow the semi-classical procedure based on the ensemble averaging described in ref. 24. It yields the master equation13
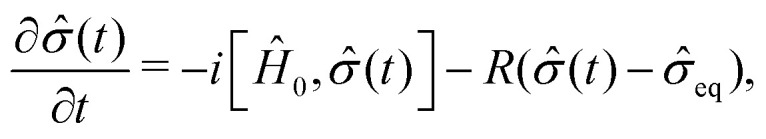
where *σ̂*_eq_ is the equilibrium density matrix given by the formula14
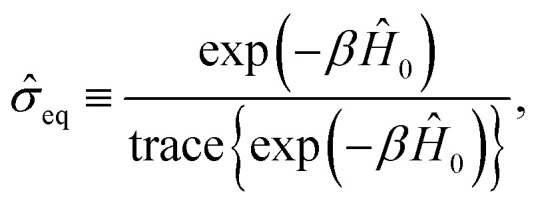
with 
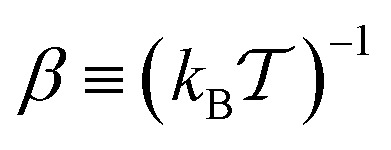
 denoting the inverse product of the system temperature, 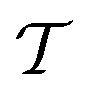, and the Boltzmann constant, *k*_B_. Eqn (13) coincides with eqn (42′′) from ref. 24, ch. VIII, if the relaxation operator is defined as15
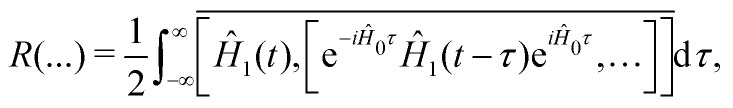
wherein the bar over the double commutator stands for the ensemble averaging. For fast isotropic rotational diffusion of nitroxides in liquids we will assume that the averaged double commutator does not depend on the absolute time *t*. Therefore we will continue the calculation of *R* with *t* = 0.^24,57^

In order to analyze experiments in which samples are irradiated by an alternating magnetic field **B**_1_(*t*) we introduce an additional term *i*[*Ĥ*_irr_(*t*),*σ̂*(*t*)] into eqn (13), which corresponds to the spin interaction with the field **B**_1_(*t*), *i.e.*,16
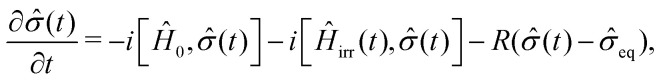
wherein

17 *Ĥ*_irr_(*t*) = −**B**_1_(*t*)(*γ*_e_**Ŝ** + *γ*_N_**Î**),

In this work we consider a linear polarized field **B**_1_(*t*) along the *x*-axis of the laboratory frame with an arbitrary magnitude, frequency and phase dependence **B**_1_(*t*) = (*B*_1_(*t*),0,0).

To proceed further with the solution and interpretation of the master eqn (16), it is rewritten in the Liouville space. We do it by constructing a vector ***σ*** out of the density matrix *σ̂*, by using the (*i*th,*j*th) density matrix element for the ((*i* − 1) ×*n* + *j*)-th component of the vector column ***σ***, where *n* is the dimension of the spin system Hilbert space. Furthermore, the equation for ***σ*** is obtained by the transformation of the commutators, which stay at the right side of eqn (16), to the Liouville space. This transformation, which is well described in the literature,^58^ yields the equation18
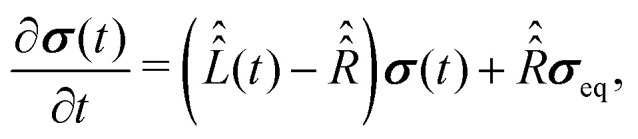
with the superoperator 
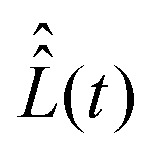
 defined as19
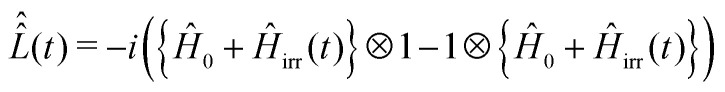
and the relaxation superoperator20
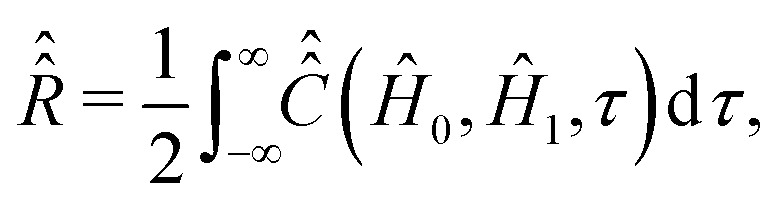
which is the time integral of a correlation superoperator, 
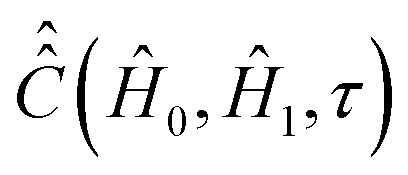
, constructed out of the Hilbert space Hamiltonian operators *Ĥ*_0_ and *Ĥ*_1_*via*21
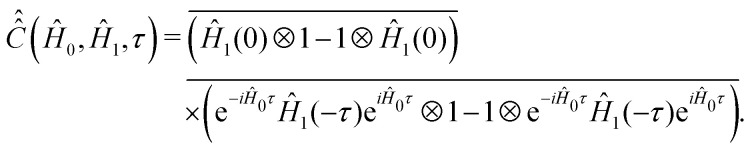
Explicitly, 
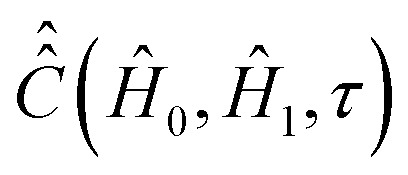
 is computed with the parameters corresponding to fast rotational diffusion in the next section.

## III. Results

### A. Solution of the master equation

Determination of vector ***σ***(*t*) from eqn (18) requires explicit values of the superoperators 
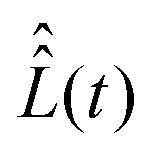
, which determines the coherent spin motion, and 
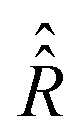
 responsible for spin relaxations. The strategy to compute these superoperators and the algorithm to solve the master equation numerically are presented in this section.

#### Computation of the superoperator 
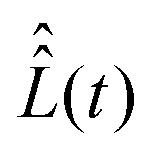


1.

The computation of the superoperator 
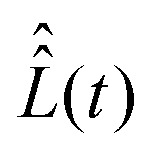
 is straightforward using expression (19), which contains the Hamiltonian operators *Ĥ*_0_ and *Ĥ*_irr_(*t*). The isotropic Hamiltonian *Ĥ*_0_ is determined using expression (10) with a given value of the static magnetic field *B*_0_ and the principal axes values of the magnetic tensors *g*^pa^ and *A*^pa^ provided in the Section II.A. The Hamiltonian *Ĥ*_irr_(*t*) requires a definition of the alternating magnetic field **B**_1_(*t*). In this work, the electron and nuclear spin magnetization are simulated by solving master eqn (18) with a linearly polarized **B**_1_(*t*) field along the *X*-axis of the laboratory frame. The magnitude of the vector **B**_1_(*t*) is assumed to have the following quite general form

22 *B*_1_(*t*) = *B*_1_ cos(*Φ*(*t*)),

with the phase *Φ*(*t*) determined by the time-dependent frequency *ν*(*t*) *via* the formula23
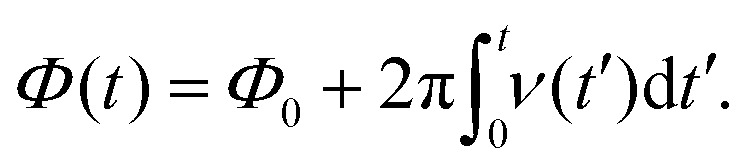
wherein *Φ*_0_ is the initial phase. For a frequency scan experiment we assume that *ν*(*t*) varies with the rate 
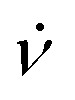
 in the interval (*ν*_m_ − Δ*ν*/2, *ν*_m_ + Δ*ν*/2) with a mean frequency *ν*_m_ and a width Δ*ν*. In this case the frequency offset δ*ν*(*t*) = *ν*(*t*) − *ν*_m_ is equal to24
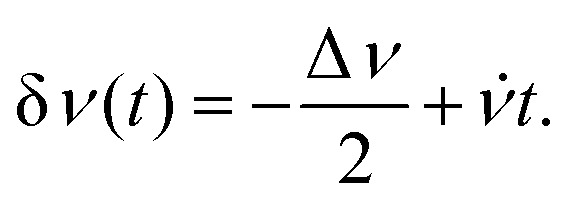
Here we consider *B*_1_(*t*) oscillating with a linearly varying frequency although other frequency dependencies can be easily implemented in our simulations. Application of a *B*_1_(*t*) field with a varied frequency corresponds to the frequency scan magnetic resonance experiments, which are being actively developed at present in EPR.^46^

#### Computation of the relaxation superoperator 
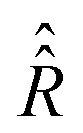


2.

In comparison to 
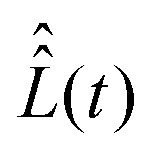
, the determination of 
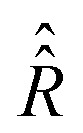
 by eqn (20) is more complicated and needs some additional assumptions. The correlation superoperator 
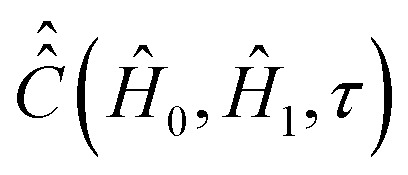
 entering integral (20) can be computed if both parts *Ĥ*_1a_(*τ*) and *Ĥ*_SR_(*τ*) of the time-dependent Hamiltonian *Ĥ*_1_(*τ*) are known exactly. However, utilization of some general properties of *Ĥ*_1a_(*τ*) and *Ĥ*_SR_(*τ*) without detailed information about them allow us to simplify the calculation of 
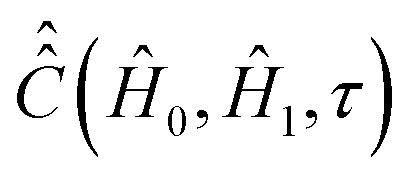
 noticeably. Eqn (12) and (7) show that *Ĥ*_1a_(*τ*) is determined by the nitroxide radical orientation **Ω**(*τ*). The spin rotation coupling term (6) depends additionally on the nitroxide angular velocity ***η***(*τ*). Using molecular dynamic simulations, it was shown that the angular velocity correlation time, *τ*^*η*^_c_, is much shorter than the rotational correlation time *τ*_c_ determined by the function **Ω**(*τ*).^51^ According to the literature,^51,59^ this allows us to neglect the cross correlation terms of *Ĥ*_1a_(*τ*) with *Ĥ*_SR_(*τ*) in the explicit form of eqn (21), which yields,25
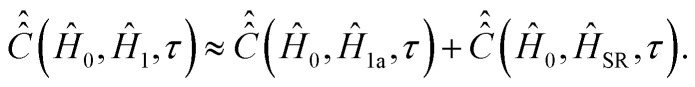
Now in order to compute the first term on the right side of the last equation we define *K* nitroxide rotation trajectories **Ω**(*τ*) and hence compute *Ĥ*_1a_(*τ*) for each trajectory explicitly. To define one **Ω**(*τ*) in a time interval (−*T*,*T*) we split this interval in 2*N* steps with a length of Δ*τ* = *T*/*N* or introduce a discrete time variable *τ*_*i*_ with *i* = −*N*, −*N* + 1,…,*N* and *τ*_−N_ = −*T*, *τ*_−N+ 1_ = −*T* + Δ*τ*, …, *τ*_N_ = *T*. The value of *T* is chosen much longer than the rotational correlation time *τ*_c_ of our liquid solved nitroxides, and *N* is large enough to make Δ*τ* much shorter than *τ*_c_. Furthermore, we assign to each time *τ*_*i*_ a nitroxide orientation **Ω**(*τ*_*i*_) = (*α*_*i*_,*β*_*i*_,*γ*_*i*_), wherein *α*_*i*_,*β*_*i*_,*γ*_*i*_ are the corresponding Euler angles. In the first trajectory, the initial orientation is set to **Ω**(0) = (0,0,0), *i.e.*, collinear to the laboratory coordinate system axes. To define other orientations **Ω**(*τ*_*i*_) we employ random number generation and iteration procedure. For each *i*, we assign an axis **n**_*i*_, which can be randomly oriented in all possible space directions with equal probability, and a normally distributed random angle *ϕ*_*i*_, with the averaged value equal to zero and dispersion *σ*_*ϕ*_. For any positive *i*, the orientation **Ω**(*τ*_*i*_) is obtained *via* a rotation of the frame **Ω**(*τ*_*i*−1_) by the angle *ϕ*_*i*_ about the axis **n**_*i*_. For negative *i*, **Ω**(*τ*_*i*_) is obtained in the same way from the frame **Ω**(*τ*_*i*+1_). This completes the definition of the first stochastic rotation trajectory with **Ω**(0) = (0,0,0).

**Fig. 2:**
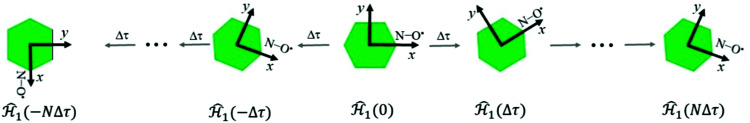
Illustration of the Monte-Carlo simulation of the random nitroxide rotations. For simplicity reasons, rotational diffusion is presented in a two dimensional picture. Hexagons with coordinate axes correspond to a TEMPOL moving along rotational trajectory with a time increment Δ*τ* from *T* = −*N*Δ*τ* to *T* = *N*Δ*τ*. At each step the nitroxide radical is rotated around a randomly chosen axis by a normally distributed angle.

The discrete trajectory **Ω**(*τ*_*i*_) defined above allows us to compute the values of the magnetic tensors *g*(*τ*_*i*_), *A*(*τ*_*i*_), and *Q*(*τ*_*i*_) using formula (7) and, thus, of the Hamiltonian *Ĥ*_1a_(*τ*_*i*_) for each time point *τ*_*i*_. Schematically the process of the *Ĥ*_1a_(*τ*_*i*_) value assignment is shown in Fig. 2. The procedure described above gives us a way to model rotational diffusion of nitroxide radicals in liquid solvents by the Monte-Carlo method. For the ensemble averaging required by expression (21), we repeat the generation of rotational trajectory *K* times by a systematic variation of the initial orientation **Ω**(0) to cover all possible directions homogeneously. In this way the value of 
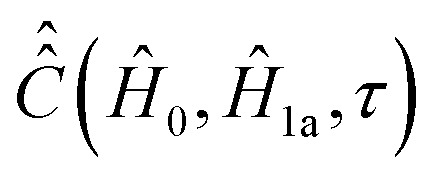
 is calculated as a function of time *τ*.

In order to demonstrate some of its properties we compute 
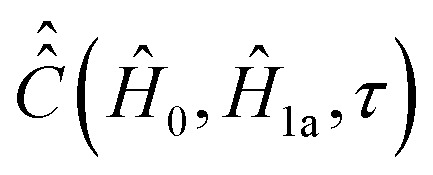
 with *Ĥ*_0_ = 0 or we set exp(−*iĤ*_0_*τ*) ≈ 1 in expression (21). This assumption can be made for very short rotational correlation times, when the *Ĥ*_0_*τ* matrix element absolute values are much smaller than the ones in the range (−*τ*_c_ < *τ* < *τ*_c_), which is typical for low *B*_0_ fields.

**Fig. 3:**
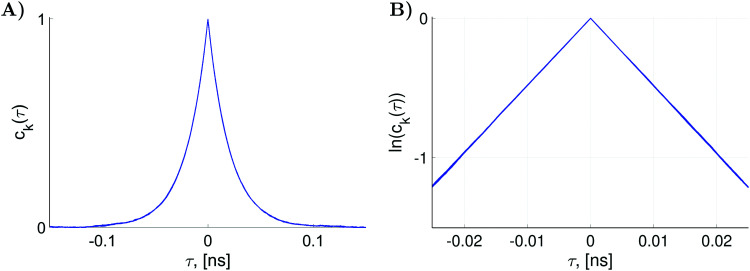
(A) The functions *c*_*k*_(*τ*) defined as the normalized diagonal elements of the superoperator 
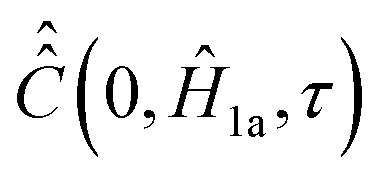
. They were simulated with *K* = 27 000 random rotational trajectories, *T* = 150 ps, Δ*τ* = 0.1 ps and *σ*_*ϕ*_ = 2.3°. (B) The plot of the functions ln(*c*_*k*_(*τ*)) which yields rotational correlation time *τ*_c_ ≈ 0.02 ns.

For nitroxide isotope ^14^*N*, the computation of 
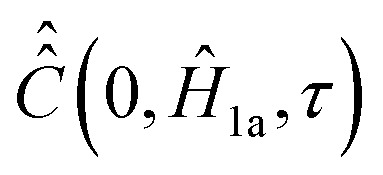
 yields a sparse 36 by 36 matrix for each time *τ*_*i*_. Fig. 3 shows time dependence of 
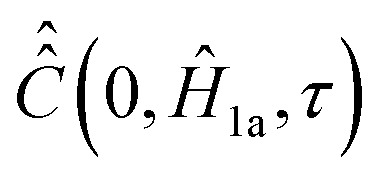
 normalized diagonal elements 
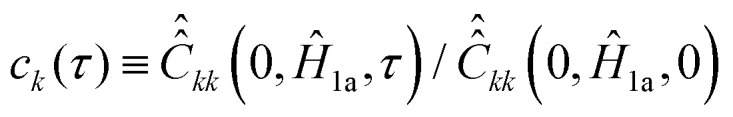
. The functions *c*_*k*_(*τ*) were computed employing randomly generated trajectories as described above with the parameter *σ*_*ϕ*_, which characterizes random rotation at each step of the trajectory generation, equal to 2.3°. For the simulations, we used the magnetic tensor principal axes values provided in Section II. A and the value of the static magnetic field *B*_0_ = 1.2 T. As shown in Fig. 3 all 36 curves of *c*_*k*_(*τ*) are almost identical. That is, they are presented virtually by one curve. This curve can be well approximated by an exponential function exp(−|*τ*|/*τ*_c_). Hence, by plotting ln(*c*_*k*_(*τ*)) the rotational correlation time, *τ*_c_, which is approximately equal to 0.02 ns in this case, is determined (see Fig. 3B).

Now we consider the second term in the expression (25), which is determined by the variation of the angular velocity ***η*** (*τ*). We assume that the 
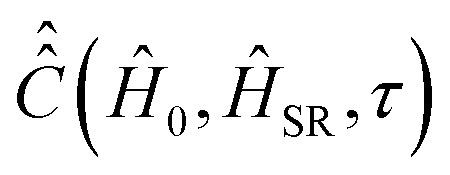
 matrix elements decay within a characteristic time *τ*^*η*^_c_, which is in the range from 10 to 100 fs as follows from molecular dynamics simulations.^51^ For such short correlation times, the matrix 
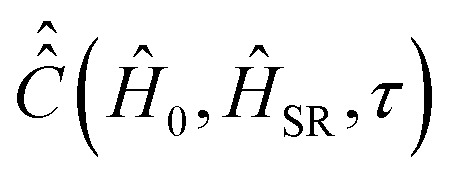
 can be substituted with 
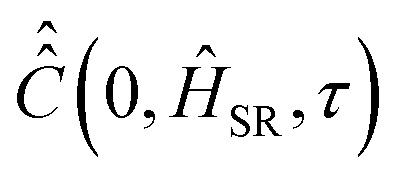
 on the time scale of *τ*^*η*^_c_ even for *Ĥ*_0_ calculated for high magnetic fields. Assuming that 
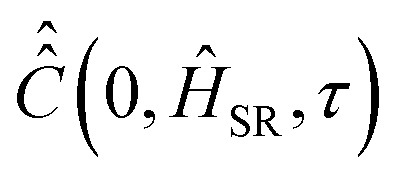
 decays exponentially, that is 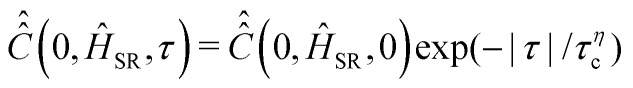, expression (20) can be transformed to26
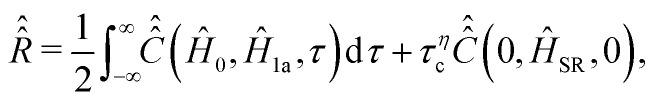
The value of 
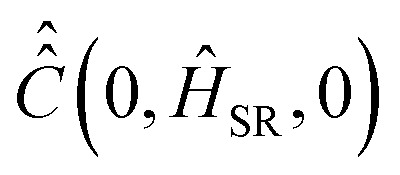
 is calculated according to (21) by the ensemble averaging of (*Ĥ*_SR_(0) ⊗ 1 − 1 ⊗ *Ĥ*_SR_(0))^2^ over all possible initial orientations of nitroxide and angular velocity ***η***(0). The absolute value of the nitroxide angular velocity is assumed to be constant throughout the ensemble and determined by the formula 
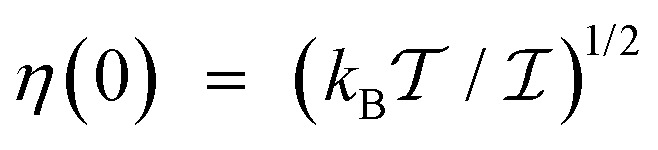
, where 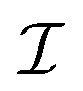 is the inertia moment of nitroxide. For TEMPOL with 
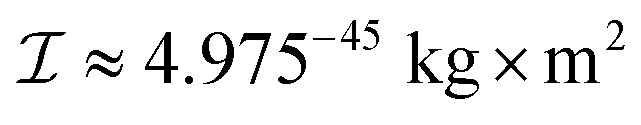
 at a temperature of 300 K, the last formula yields *η*(0) ≈ 9.125 × 10^11^ rad s^−1^. The strategy to compute the relaxation superoperator described above is used to determine the electron and nuclear spin dynamics of nitroxide in the next sections.

From the above described algorithm of the relaxation superoperator determination, it follows that 
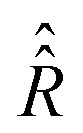
 depends among other parameters on *σ*_*ϕ*_ and *τ*^*η*^_c_, which characterize random nitroxide molecular motion and are influenced by solvent viscosity and temperature in real samples. This relation allows us to account for solvent and temperature effect in our simulation. For instance, the utilization of a lower viscosity solvent would correspond to an increase in parameters *σ*_*ϕ*_ and *τ*^*η*^_c_, whereas a higher viscosity solvent would require a decrease of these parameters. Similarly, heating and cooling of the sample can be modeled by the increase and decrease of *σ*_*ϕ*_ and *τ*^*η*^_c_, respectively.

#### 3. Numerical solution of the master equation

In order to solve the master eqn (18), it is integrated over time window (*t*_0_,*t*_0_ + Δ*t*). This yields the following equation27
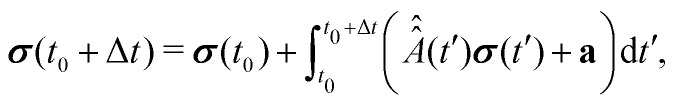
wherein 
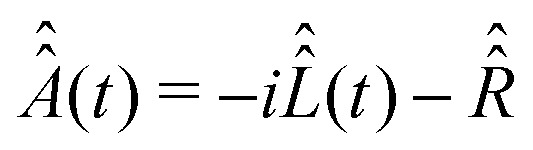
 and 
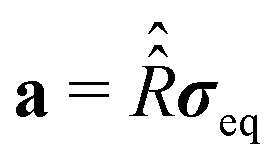
. For our purpose, the time increment Δ*t* is chosen short enough to neglect the variation of the superoperator 
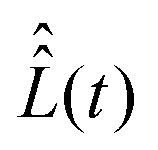
 within it. This can be achieved when Δ*t* is much shorter than the *B*_1_(*t*) oscillating period 
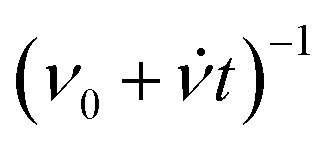
. Assuming that the superoperator 
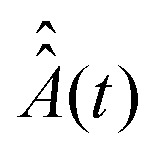
 is constant within the time (*t*_0_,*t*_0_ + Δ*t*), the value of the vector ***σ*** at the time point *t*_0_ + Δ*t* can be approximately found from ***σ*** (*t*_0_) by applying formula (27) iteratively. This gives us the following expression,28
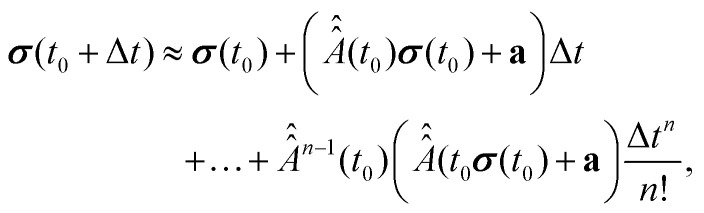
with *n* denoting the number of iterations or the order of precision. Obviously, an increase of *n* or reduction of Δ*t* will increase the precision of ***σ***(*t*), but will extend the computational time. In this work, Δ*t* and *n* were chosen large and small enough, respectively, that further increase of *n* or decrease of Δ*t* did not make any visible changes in the simulated signals.

Using eqn (28), ***σ*** can be computed for any time *t* by doing enough Δ*t* steps starting from zero time and the equilibrium density matrix. For instance, in order to simulate the CW-EPR spectrum, ***σ***(*t*) is computed for the time 
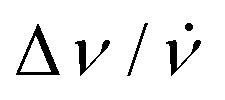
 wherein Δ*ν* stands for the frequency range necessary to record this spectrum. Furthermore, the vector ***σ***(*t*) is transformed back to the Hilbert space density matrix *σ*(*t*), which is used to compute spin magnetization **M**(*t*) *via* the formula

29 **M**(*t*) = trace(**M̂***σ*(*t*))

where **M̂** is the operator of the spin magnetic moment equal to *γ*_e_*ħ***Ŝ** or *γ*_N_*ħ***Î** for electron and nitrogen nucleus, respectively. Expression (29) gives values of the spin magnetization in the laboratory frame. In order to obtain the transverse magnetization value in the frame rotating with the time-dependent phase *Φ*(*t*) around the *z*-axis of the laboratory frame, **M**(*t*) is transformed *via* the formula

30 *m*_*x*,(*y*)_(*t*) = Re(Im){e^−*iΦ*(*t*)^(*M*_*x*_(*t*) + *iM*_*y*_(*t*))}.

For a CW-EPR experiment with linearly varied frequency, the magnetization values *m*_*x*_ and *m*_*y*_ can be plotted *versus* the frequency *ν* or the frequency offset δ*ν* yielding the usual adsorption and dispersion spectra.

### B. Electron spin dynamics of liquid solved nitroxides

The EPR spectra of organic radicals and particularly of nitroxides solved in liquids were already studied for a long time. Their dependence on various experimental and system parameters is well understood and modeled.^33^ The effects of the static and microwave magnetic fields, temperature and stochastic rotational molecular motion on the spectral form are well known. Based on this knowledge, we will rather evaluate and calibrate our simulation procedure for later use in the computations of nitroxide nuclear spin dynamics then improve the performance of the established methods for the simulation of EPR spectra.

In order to simulate spin magnetization according to the computational strategy described above, a self-written MATLAB program was used. The input parameters of this program are summarized below.

• Temperature: 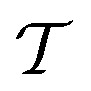.

• The value of the static magnetic field: *B*_0_.

• Parameters characterizing alternating magnetic field: *B*_1_, *Φ*_0_, *ν*_0_, Δ*ν* and 
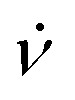
.

• Values of the magnetic tensors in the principal axes frame: *g*^pa^, *A*^pa^, *Q*^pa^.

• Parameters for the computation of the relaxation superoperator 
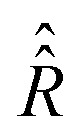
 from the random rotational trajectories: *K*, *σ*_*ϕ*_, Δ*τ*, 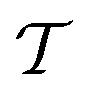, 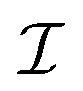 and *τ*^*η*^_c_.

• Time increment Δ*t* and the precision order *n* for the propagation of ***σ***(*t*).

**Fig. 4:**
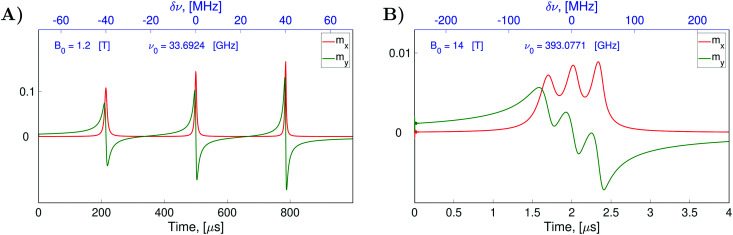
Simulated evolution of the electron spin magnetization of liquid solved nitroxides in CW-EPR experiments with varied MW frequencies and fixed static magnetic fields *B*_0_ = 1.2 T in (A) and *B*_0_ = 14 T in (B). The parameters of the MW field were chosen as *B*_1_ = 2 × 10^−5^ T, *Φ*_0_ = π/2 and 
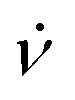
 = 140 GHz s^−1^ for (A) and *B*_1_ = 2 × 10^−5^ T, *Φ*_0_ = π/2 and 
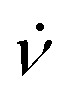
 = 125 THz s^−1^ for (B). The relaxation superoperator 
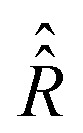
 was determined with *K* = 27 000, *T* = 150 ps, Δ*τ* = 0.1 ps, *σ*_*ϕ*_ = 2.3°, 
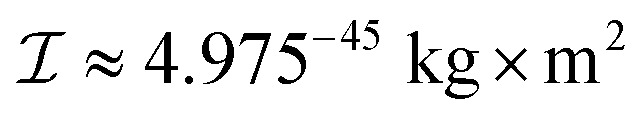
, *τ*^*η*^_c_*=* 30 fs and *T* = 300 K in both cases (A and B). Eqn (28) was solved with Δ*t* = 1 ps and *n* = 8 in (A) and Δ*t* = 0.02 ps and *n* = 8 in (B).

For the demonstration of the program performance, we simulated frequency scan EPR spectra at a low magnetic field of 1.2 T and a high field of 14 T (see Fig. 4). Parameters *B*_1_ and *Φ*_0_ characterizing the MW field were constant in both cases. The central frequency *ν*_0_ of the scanned frequency range (*ν*_0_ − Δ*ν*/2, *ν*_0_ + Δ*ν*/2) was tuned to the frequency of the central nitroxide EPR line in each case. The frequency scan rate 
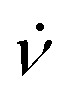
 was chosen according to the condition 
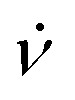
 ≪ *T*_2_^−2^ to avoid the appearance of non-stationary effects in the computed spectra. The parameters of radical random rotational motion (*σ*_*ϕ*_ and Δ*τ*) were also set equal in both simulations. By choosing *σ*_*ϕ*_ = 2.3° and Δ*τ* = 0.1 ps for the generation of nitroxide random rotational trajectories, we obtained *τ*_c_ = 20 ps. This value is in a good agreement with the rotational correlation times of the water solved TEMPOL at room temperature obtained by molecular dynamic simulations and by fitting of the CW-EPR spectra.^51,62^

**Fig. 5:**
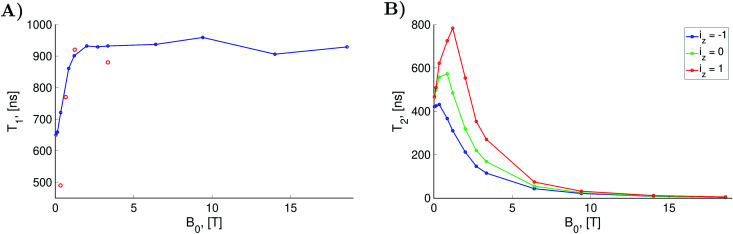
(A) Computed (doted blue line) and experimental (red circle) longitudinal electron spin relaxation times as functions of the static magnetic field *B*_0_. The shown experimental data are for TEMPONE in water at 20 °C.^51,60,61^ (B) Transverse relaxation times for the three nitroxide hyperfine lines. The computations were performed with the relaxation superoperator 
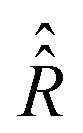
 determined by the dynamical parameters corresponding to the rotational diffusion motion of TEMPOL in water which are specified in the caption of Fig. 4.

Fig. 4 shows a significant broadening of the EPR line at high magnetic fields. This is attributed to the shortening of the *T*_2_ relaxation time by high magnetic fields, which increase the effects of the *g*-tensor anisotropy. Using simulated *m*_*x*_(*ν*) and *m*_*y*_(*ν*) we determined the electron spin transverse relaxation times for each hyperfine line of the ^14^*N*-nitroxide EPR spectrum with nitrogen nuclear spin projection *i*_*z*_ equal to −1, 0 or 1. To this end we employed the formula31
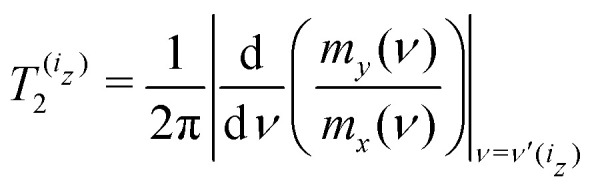
which can be obtained from the stationary solution of the Bloch equation.^24^ In formula (31), *ν*′(*i*_*z*_) = *ν*_0_ + *i*_*z*_*A*_0_/(2π) corresponds to the spectral position of the hyperfine line with the nuclear spin quantum number *i*_*z*_. In this way, we estimated *T*_2_ times from the spectra for *B*_0_ equal to 1.2 T and 14 T shown in Fig. 4A and B and also from the frequency scan EPR spectra simulated with other *B*_0_ values in the range from 0.035 T to 18.6 T shown in Section S1 of the ESI.† The obtained *T*_2_ values for the three hyperfine lines are shown in Fig. 5 as a function of the static magnetic field.

In frames of our computational model, we determined electron spin *T*_1_ relaxation times for the same range of *B*_0_ fields. For this purpose, we computed the recovery of longitudinal electron spin magnetization after a 5 μs long MW pulse with the frequency fixed at the central nitroxide line and with a magnitude of 0.2 mT. The length of the pulse was long enough for the magnetization to almost reach their stationary values. Assuming the exponential form for the decay of *M*_eq_ − *M*_*z*_(*t*) the *T*_1_ relaxation times were determined (see Fig. 5A). Details of the longitudinal electron spin magnetization simulations, as well as the estimation of *T*_1_ times from the simulated data, are presented in the ESI† (see S2).

The frequency scan CW-EPR spectra and the relaxation times, which were simulated in this section, exhibit the most important spectral characteristics of fast rotating nitroxides solved in liquids and agree well with the known experimental data. A more detail comparison of the nitroxide spectra computed using our computational approach based on direct solution of the LvN equation to the experimental data and spectra computed by other EPR computational tools (*e.g.* EasySpin) is presented in the ESI† (see S3 and S4). With this calibration of our computational method, we proceed to the simulation of nitroxide nitrogen nuclear spin magnetization.

### C. Nitrogen nuclear spin dynamics

The main goal of this work is to gain insights into the nitrogen nuclear spin dynamics of fast rotating nitroxide radicals in liquid solutions. To complete this task, we will employ the nitroxide spin Hamiltonian specified in the Section II.A and the model for the molecular rotational diffusion described in the Section III.A. Also, we will use the computational method introduced above which can deliver accurate EPR spectra and relaxation times for nitroxides in a broad frequency range. However, before doing detailed computation, we infer some essential characteristics of the nitrogen nuclear spin dynamics qualitatively.

In nitroxide molecules, the nitrogen nuclear spin is in close vicinity of the unpaired electron spin, which produces a strong magnetic field *B*_e_ at the nitrogen nucleus position. The characteristic magnitude of this field can be estimated as the energy, which is introduced by the isotropic hyperfine coupling Hamiltonian term *A*_0_**ŜÎ**, divided by the nitrogen nuclear spin magnetic moment, that is *B*_e_ = *A*_0_/(2*γ*_N_). With the hyperfine tensor values defined in Section II.A, we obtain *B*_e_ ≈ 6.5 T. Interestingly, this value of the magnetic field is higher than the static magnetic field of common EPR spectrometers but lower than *B*_0_ of popular NMR spectrometers.

**Fig. 6:**
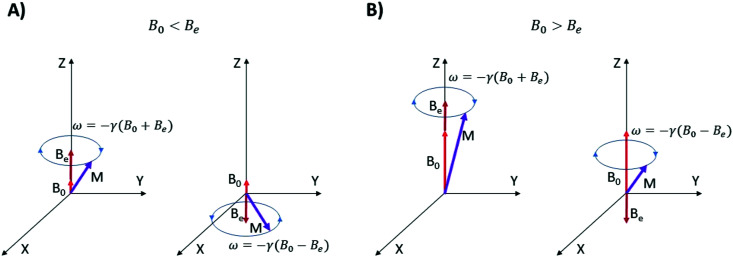
Precession angular velocities of nitroxide nitrogen nuclear magnetic moments in an effective magnetic field consisting of the external field *B*_0_ and the contribution from the unpaired electron which can be positive (+*B*_e_) or negative (−*B*_e_). In (A and B) the cases *B*_0_ < *B*_e_ and *B*_0_ > *B*_e_ are illustrated, respectively.

The orientation of *B*_e_ is determined by the orientation of the unpaired electron spin (Fig. 6). This means that nitrogen nuclei in nitroxides with electron spin oriented along *B*_0_ are exposed to a higher magnetic field *B*_0_ + *B*_e_ and the nitrogen nuclei in nitroxides with the anti-parallel electron spin orientation are in the lower field *B*_0_ − *B*_e_. Since the numbers of electron spins up and down are almost equal at high temperatures, we can split the ensemble of nitroxides into two nearly equal sub-ensembles that have effective magnetic fields at the positions of nitrogen nuclei *B*_0_ + *B*_e_ and *B*_0_ − *B*_e_, respectively. In the classical picture, the nitrogen nuclear magnetic moments precess in these fields with angular velocities of −*γ*_N_(*B*_0_ + *B*_e_) and −*γ*_N_(*B*_0_ − *B*_e_), respectively. Hence, we expect two lines split by the frequency gap 2*γ*_N_*B*_e_/(2π) in the NMR spectrum of nitroxide radicals.

**Fig. 7:**
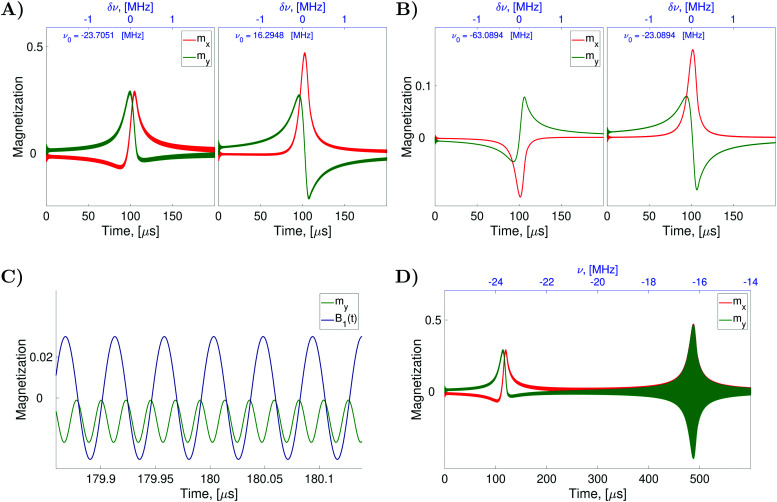
Simulated nitrogen NMR spectra of liquid solved nitroxides. The plots (A and B) show the evolution of the transverse nuclear magnetization in CW-NMR experiments with varied radio frequencies and fixed static magnetic fields *B*_0_ = 1.2 T in (A) and *B*_0_ = 14 T in (B). The radio frequency field is defined with *B*_1_ = 1 × 10^−3^ T, *Φ*_0_ = 0 for all simulations. The centers (parameter *ν*_0_) and the width of the scanned frequency ranges as well as the scan time are indicated in the plots individually. As in the previous two figures (see Fig. 4 and 5) the computations were performed with the relaxation superoperator 
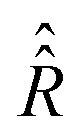
, which is determined by the dynamical parameters corresponding to the rotational diffusion motion of TEMPOL in water, which are specified in the caption of Fig. 4. Eqn (28) was solved with Δ*t* = 5 ps and *n* = 128 in all simulations. The plots (C and D) are the zoom in and the zoom out, respectively, of the left side plot of the plot (A).

To verify the qualitative consideration presented above and to describe nitrogen NMR lines quantitatively, we simulated frequency scan experiments on liquid solved nitroxides. As in the case of the EPR spectra demonstrated in the previous section, we performed simulations with the static magnetic fields of 1.2 T and 14 T (see Fig. 7A and B). For each *B*_0_ value, two frequency ranges with a width of 4 MHz were scanned. The central points of the first and the second regions were chosen to be equal to 
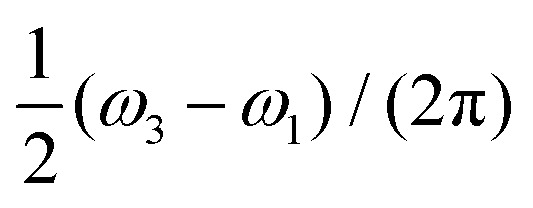
 and 
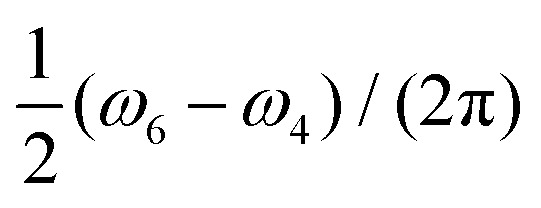
, respectively, wherein *ω*_*i*_ with *i* = 1,2,…,6 are eigen values of the isotropic Hamiltonian *Ĥ*_0_ given by formula (10). The magnitude, the initial phase, and the frequency scan rate of the radio frequency magnetic field *B*_1_(*t*) were kept equal in all simulations and are specified in the figure caption. The relaxation operators 
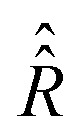
 at *B*_0_ values of 1.2 T and 14 T were taken the same as those which were used for the simulation of the corresponding EPR spectra (see Fig. 4A and B). With these settings, the computation of nuclear spin magnetization as a function of time or radio frequency, which is linearly linked to the time in the frequency scan experiment, was performed in the laboratory frame. Furthermore, the laboratory frame signal was transferred to the rotating frame *via* formula (30). As predicted qualitatively, we observe resonance behaviors of the nuclear spin magnetization or NMR lines at two frequencies, which roughly correspond to the nitrogen nuclear spin precession angular velocities in the magnetic fields *B*_0_ − *B*_e_ and *B*_0_ + *B*_e_. Also, as expected, we obtained a large by NMR standards line width caused mainly by the strong influence of the unpaired electron on the nitrogen spin (half-width at half-height is about 100 kHz).

However, a closer look at the spectral lines shown in Fig. 7A and B poses questions, which do not have immediate answers. The first striking thing which is seen in Fig. 7A, is the presence of relatively small oscillations at high frequency superimposed upon a relatively large slowly changing signal associated with the NMR lines. Due to these oscillations, the curves shown in the Fig. 7A appear bolder. To explain the origin of the high frequency oscillations, we zoom-in and zoom-out Fig. 7A. By zooming-in the curve *m*_*y*_(*t*) (see Fig. 7C), we observe that the frequency of the fast oscillations is precisely double the frequency of *B*_1_(*t*), which is also plotted in arbitrary units in Fig. 7C in blue color. By zooming-out the curve *m*_*y*_(*t*) (see Fig. 7D), we observe an increase in the magnitude of the high frequency oscillations until −16.2948 MHz, which is the negative value of the peak frequency *ν*_0_ of the right side plot in Fig. 7A. As discussed above the peak at −23.7051 MHz shown on the left side of Fig. 7A is assigned to the nitroxide subensemble with the nitrogen nuclear spin precession velocity −*γ*_N_(*B*_0_ + *B*_e_) and the peak at 16.2948 MHz shown on the right side corresponds to the other subensemble with the precession velocity −*γ*_N_(*B*_0_ − *B*_e_), (note that *B*_0_ = 1.2 T and *B*_e_ ≈ 6.5 T in this case). Also, we remind that our simulations were performed with a linearly polarized field *B*_1_(*t*), which can be presented as two circularly polarized fields rotating in opposite directions with the angular velocities of 2π*ν*(*t*) and −2π*ν*(*t*), respectively. Hence, the *B*_1_(*t*) field with a frequency *ν*(*t*) close to −16.2948 MHz can excite not only nitrogen nuclear spins with a precessing velocity of −2π × 16.2948 Mrad s^−1^ but also nitrogen nuclear spins with a precessing velocity of 2π × 16.2948 Mrad s^−1^. Since the signal shown in Fig. 7A is the transverse nuclear spin magnetization in the frame rotating with an angular velocity of 2π*ν*(*t*), the nuclear spin precession with an angular velocity of −2π*ν*(*t*) gives rise to the observed signal at the double frequency. The effect described above is well pronounced when *B*_0_ ≪ *B*_e_. In this case, the two circular components of the *B*_1_(*t*) field, which rotate with angular velocities of 2π*ν*(*t*) and −2π*ν*(*t*), can simultaneously match with the precessing angular velocities −*γ*_N_(*B*_0_ + *B*_e_) and −*γ*_N_(*B*_0_ − *B*_e_) of the first and the second nitroxide subensemble, respectively. The curves, which are shown in Fig. 7B obtained for *B*_0_ = 14 T which does not satisfy the condition *B*_0_ ≪ *B*_e_, do not exhibit high frequency oscillations. In a possible experiment, however, the observation of the double frequency oscillations can be eliminated by the spectrometer's low-pass filtering.

Another striking fact of the spectra shown in Fig. 7A and B is the following. In the simulation of these spectra, we used *B*_1_(*t*) with the initial phase *Φ*_0_ = 0, which corresponds to the *B*_1_ field orientation along the *x*-axis of the rotating frame. For simple isolated spin magnetic moments, such a field should produce a Lorentzian shape symmetric *y*-component of nuclear spin magnetization *m*_*y*_(*ν*) in the rotating frame. However, the curves *m*_*y*_(*ν*) shown in green in Fig. 7A and B do not exhibit such form. In Fig. 7A and B the curves *m*_*x*_(*ν*) look rather as Lorentzian functions. Here, we explain such a considerable distortion of the nitroxide nitrogen NMR line shape by the interaction of the unpaired electron spin with the radio frequency field *B*_1_(*t*), which is described by the Hamiltonian (17). Although the radio frequency is far off-resonant for electron spins, it causes small oscillations of electron spin magnetization, which affect nitrogen nuclear spin dynamics due to hyperfine coupling. To verify this statement, we performed the simulations of the spectra with the same parameters except for the Hamiltonian term *Ĥ*_irr_(*t*), which was simplified in comparison to (17) just to account for the interaction of the nitrogen nuclear spin with the radio frequency magnetic field, that is *Ĥ*_irr_(*t*) = −*γ*_N_**B**_1_(*t*)**Î**. With this ‘switch off’ of the electron spin interaction with the radio frequency irradiation, the shapes of *m*_*x*_(*ν*) and *m*_*y*_(*ν*) curves get their usual form (see ESI,† S5).

As further investigation of the nitrogen nuclear spin magnetization, we computed population of the nitroxide spin system energy levels subjected to irradiation with a microwave frequency exciting an electron spin transition. We analyzed the population 
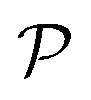
 of all six nitroxide ^14^*N* spin states, 
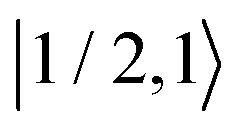
, 
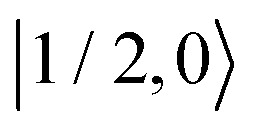
, 
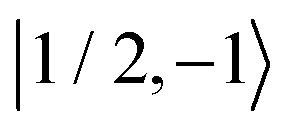
, 
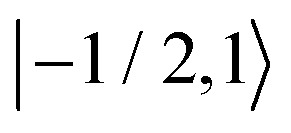
, 
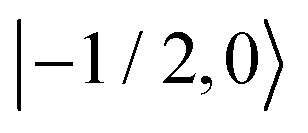
, and 
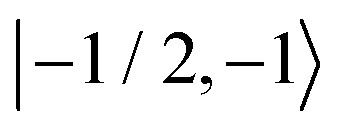
, as a function of time, when the central nitroxide EPR line was irradiated. These populations were obtained as diagonal elements of the spin density matrix computed by our numerical approach and allowed us to determine the longitudinal nitrogen nuclear spin and related to it electron spin magnetization straightforwardly. According to eqn (29), the longitudinal nitrogen nuclear magnetization is proportional to 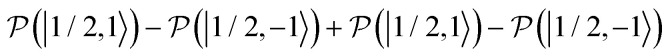 and the longitudinal electron spin magnetization is proportional to 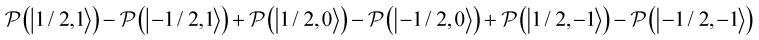.

**Fig. 8:**
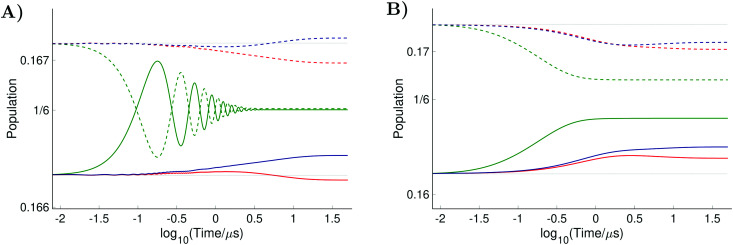
Evolution of the spin state population upon microwave irradiation of the central nitroxide EPR line for *B*_0_ = 1.2 T in (A) and for *B*_0_ = 14 T in (B). The red, green and blue solid (dashed) curves correspond to the spin states 
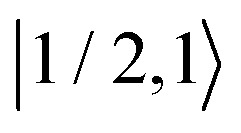
, 
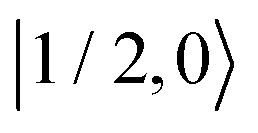
 and 
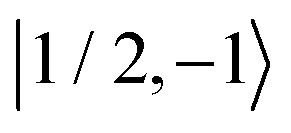
 (
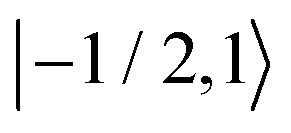
, 
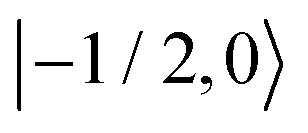
 and 
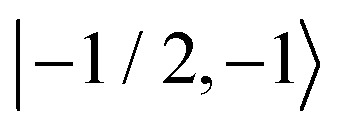
), respectively. The thin gray dotted upper and lower lines indicate initial population 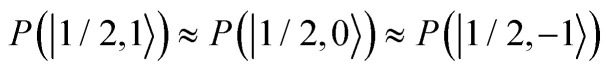 and 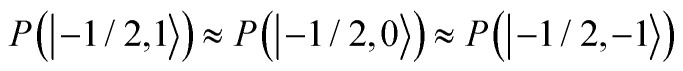, respectively.

**Fig. 9:**
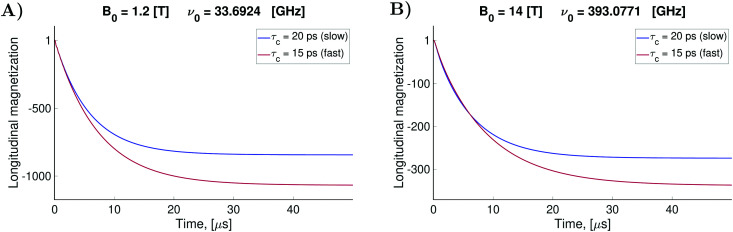
Simulated nitroxide nitrogen DNP in liquid solution. The figures show the evolution of the normalized longitudinal nitrogen nuclear spin magnetization under the constant irradiation of the central nitroxide EPR transition. Simulation was performed with *B*_0_ = 1.2 T in (A) and *B*_0_ = 14 T in (B). The frequency *ν*_0_ of the irradiating MW for each *B*_0_ value is indicated in the corresponding plots. *B*_1_ = 0.0002 T was used for all simulations. Simulations shown by the blue curves in (A and B) were performed with the same relaxation operator as was used for the simulations of the corresponding EPR and NMR spectra. The brown curves were obtained with another relaxation operator which was computed for a faster rotational diffusion process characterized by *σ*_*ϕ*_ = 2.7° and *τ*_*η*_ = 25 fs.

For the computation of the nitroxide spin system response to the irradiation of its central EPR line, the microwave field parameters Δ*ν*, 
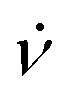
, and *Φ*_0_ were set to zero. The magnitude of the laboratory frame linearly polarized field *B*_1_(*t*) was fixed at 0.0002 T. As before, we chose for these simulations a low static magnetic field *B*_0_ = 1.2 T typical for EPR technique and a high *B*_0_ = 14 T, which is often used in NMR spectrometers. With the defined magnetic fields we computed the nitroxide spin density matrix and the spin magnetization by solving the master eqn (18) using the same relaxation operator as for the computation of the corresponding EPR and NMR spectra shown above. The results of the spin state population computation are presented in Fig. 8. As we see in the figure, the population of the three states with the electron spin projection 
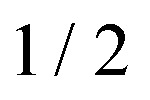
 and 
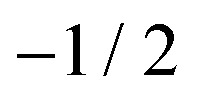
 were almost equal before the irradiation, *i.e.*, 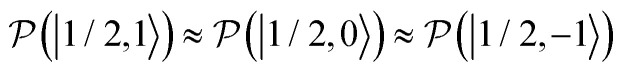 and 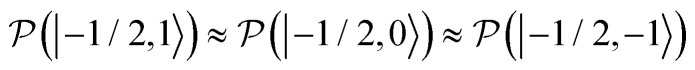. After a long irradiation on a time scale of 1 to 10 μs, the population of all spin states reaches new stationary values. This rearrangement of the spin state population increases the population differences 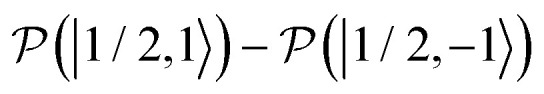 and 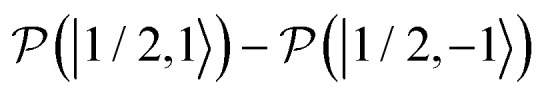, causing a large increase in the absolute value of the nitrogen nuclear spin magnetization in comparison to the equilibrium value (see Fig. 9A and B). This simulation demonstrates a quantitative prediction of the nitrogen DNP effect caused by the microwave irradiation of nitroxide unpaired electron spin. By defining the DNP enhancement factor as the ratio *ε* = (*M*^N^_*z*_(∞) − *M*^N^_*z*_(0))/*M*^N^_*z*_(0), wherein *M*^N^_*z*_(0) and *M*^N^_*z*_(∞) are the initial and stationary longitudinal nitrogen nuclear spin magnetization, we detected *ε* ≈ −844 and *ε* ≈ −275 for *B*_0_ = 1.2 T and *B*_0_ = 14 T, respectively.

Using similar simulation as those shown in Fig. 9A and B, we estimated the longitudinal nitrogen nuclear spin magnetization relaxation times for our liquid solved nitroxide radicals. For *B*_0_ = 1.2 T and *B*_0_ = 14 T, we obtained *T*_1_ ≈ 6.7 μs and *T*_1_ ≈ 7.0 μs, respectively. The details of this simulation are given in the ESI† (see S6).

Additionally, we investigated the influence of radical rotational diffusion intensification, which can correspond to a temperature elevation or usage of solvents with lower viscosity, on the observed DNP effect. For this purpose, the relaxation operator was computed with faster changing random rotational trajectories in comparison to the previous simulations. We set *σ*_*ϕ*_ = 2.7°, which yields the rotational correlation times of about *τ*_c_ = 15 ns, and *τ*^*η*^_c_ = 25 fs. Such parameter variation corresponds roughly to a temperature rise by 10 K. As shown in Fig. 9 an even larger DNP effect was predicted for faster moving radicals.

According to the literature, liquid DNP enhancement *ε* can be expressed *via* the formula,32
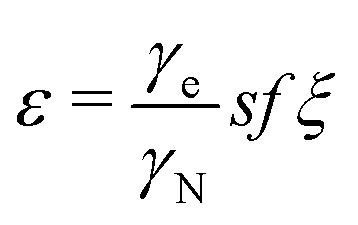
where *f* = (*b*_R_ − *b*_0_)/*b*_R_ is the leakage factor, which is determined by the longitudinal nuclear spin relaxation rate in the presence (*b*_R_) and the absence (*b*_0_) of radicals in the solution.^63^ Here, we assumed *f* ≈ 1, since the relaxation of nitrogen nuclear spin, which is caused by the very close unpaired electron in nitroxide, is much stronger than other nuclear spin relaxation mechanisms. In formula (32), *ξ* is the coupling factor reflecting the nature of the polarisation transfer between the electron and nuclear spins and *s* is the saturation factor showing the change of the longitudinal electron spin magnetization relatively to its initial value, *M*^e^_*z*_(0), that is *s* = (*M*^e^_*z*_(0) − *M*^e^_*z*_(∞))/*M*^e^_*z*_(0), where *M*_*z*_(∞) denotes the stationary value of the electron spin magnetization under microwave irradiation. Using the spin density matrices that were used for the computation of the level population in Fig. 8 and for the nitrogen nuclear magnetization in Fig. 9, we determined the saturation factors for the nitroxide randomly rotating with *τ*_c_ = 20 ps and *τ*_c_ = 15 ps in magnetic fields *B*_0_ = 1.2 T and *B*_0_ = 14 T. The results of this computation are presented in Table 1. A special attention deserves the obtained saturation factors 0.405 and 0.39 for the magnetic field value *B*_0_ = 1.2 T. They are significantly larger than 1/3, the value which would be expected in the case when only one of the three nitroxide narrow lines is irradiated with our *B*_1_ much smaller than the line splitting. This deviation can be explained by the spin state level population dynamics shown in Fig. 8A. There we see that the population difference 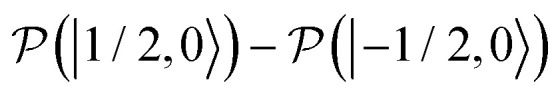, which is one of the three equal contributions, 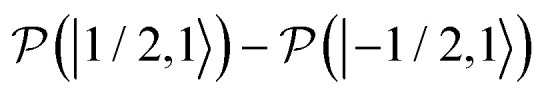, 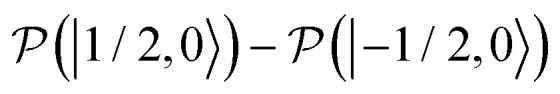, 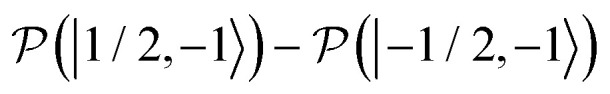, to the total longitudinal electron spin magnetization, vanishes, *i.e.*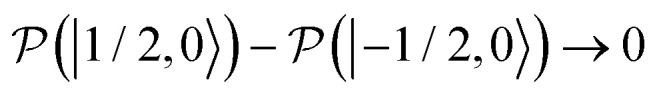. The other two contributions 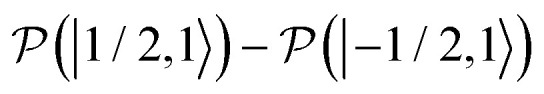 and 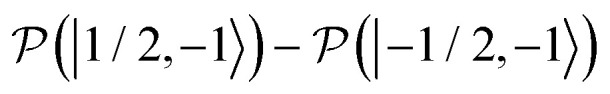 decrease visibly due to the nitrogen DNP of nitroxides yielding a saturation factor larger than 1/3. Hence, the effects of the nitrogen nuclear spin dynamics cause about 20% larger electron spin saturation factor in comparison to the saturation factor obtained treating three nitroxide lines as independent.

**Table 1:** DNP enhancement (*ε*), coupling (*ξ*), and saturation (*s*) factors obtained from the simulated nitroxide nitrogen nuclear and electron spin longitudinal magnetization for varied *B*_0_ and intensity of rotational diffusion

	*B*_0_ = 1.2 T		*B*_0_ = 14 T	
	*τ*_c_ = 20 ps (slow rotation)	*τ*_c_ = 15 ps (fast rotation)	*τ*_c_ = 20 ps (slow rotation)	*τ*_c_ = 15 ps (fast rotation)
*ε*	−844	−1069	−275	−338
*s*	0.405	0.390	0.444	0.463
*ξ*	0.229	0.300	0.068	0.080

Using simulated longitudinal nitrogen nuclear magnetization shown in Fig. 9 and the electron spin magnetization shown in the ESI† (S5), the enhancement and saturation factors were obtained, respectively. Furthermore, using formula (32), the coupling factors were determined from these simulation data. The results of these estimations are summarized in Table 1.

## IV. Conclusions and outlooks

Here we made a quantitative prediction and analysis of the nitroxide nitrogen nuclear spin dynamics in EPR and NMR experiments. Our computations were performed for liquid solved nitroxides undergoing random rotational motion, which leads to spin relaxation. We performed most of our simulations employing rotational diffusion parameters corresponding to TEMPOL radicals in water at 300 K. However, simulations of other organic radicals and solvents can be easily accomplished by repeating the simulations with carefully chosen parameters describing radical rotational motion and magnetic tensors.

Nitrogen nuclear and electron spin dynamics were determined from the nitroxide spin system density matrix obtained by solving the master equation accounting for electron and nuclear spin relaxation and for irradiating magnetic field *B*_1_(*t*). We repeated the computation for several *B*_0_ values to characterize nitrogen nuclear magnetization in a broad range of static magnetic fields. For two of them, 1.2 T, which is the field of a Q-band EPR spectrometer, and 14 T, which is in a 600 MHz proton NMR spectrometer, the results are presented and analyzed in the main text. Although the central question of this investigation was related to the behavior of the nitrogen nuclear spin, we also computed the electron spin dynamics, which was used to verify the performance of our computational approach. We performed all our simulations for fast rotating nitroxide molecules. Here, it should be mentioned that nitroxide spin labels and their EPR spectra are often used to investigate complex dynamics of large macromolecules, which do not often rotate fast enough to reach the fast motion regime. Application of our simulation method in this case of slowly rotating nitroxides would require solution of SLE with our computational strategy which has not been implemented yet. Generally, our work aims to gain additional insights into and to optimize liquid state DNP performed using nitroxide radicals which are often treated in fast rotation limit. Also, our determination of nitrogen nuclear spin behavior *via* a direct solution of the LvN equation shed additional light on the nitroxide spin dynamics. Remarkably, the solution of the master equation did not require a transition to the rotating frame or high field approximation for the spin Hamiltonian, which are frequently used in the magnetic resonance.

The MATLAB program, which we developed based on the direct solution of the LvN equation described in the manuscript and used for our computation, can be obtained for further research by contacting the corresponding author individually. The program computational time depends on the spin system and experimental parameters and can extend over several hours.

Besides the successful implementation of the computational approach to determining nitrogen nuclear spin dynamics, we would like to emphasize this research's rather methodological importance than the groundwork for experiments. Experimental detection of nitroxide nitrogen NMR would probably be complicated by weak signals. The weakness of the nitrogen nuclear signal is caused first of all by the small nitrogen nuclear gyromagnetic ratio, which is about 9100 smaller than *γ*_e_. For comparison, for protons, which are usually more abandon in samples, *γ*_e_/*γ*_p_ ≈ 660. Additionally, observation of nitrogen nuclear spin magnetization is hindered by a large NMR line width and short relaxation times caused by the strong effect of the unpaired electron spin. However, the theoretical investigation carried out here answers an interesting question of what nitrogen nuclear spin magnetization (although small in its absolute value) does in several magnetic resonance experiments. Special attention deserves the quantitative prediction of the longitudinal nitrogen nuclear spin magnetization enhancement upon microwave irradiation of the electron spin transition. It demonstrates a large nitrogen DNP effect sensitive to the magnitude of the microwave field (see ESI,† S8) and to the intensity of nitroxide rotational diffusion in solutions.

Although our current work is devoted to the analysis of nitrogen spin dynamics, the computational method, which we used, is open for further modifications, which can enable quantitative description of other nuclear spins such as ^1^H, ^2^H and ^13^C, which can be present in nitroxide radicals. As a further development, the nitroxide spin system can be extended with solvent nuclei in order to investigate the role of nitroxide nuclei in transferring electron spin polarization to solvent nuclei. Using our method, electron and nuclear spin magnetization can be computed with an arbitrary form of the *B*_1_(*t*) field. This allows us to study nuclear spin polarization using frequency modulated microwaves and describe electron spin dynamics in rapid frequency scan EPR experiments.^46,64,65^

## Conflicts of interest

There are no conflicts to declare.

## Supplementary Material

CP-023-D0CP06071B-s001
