# Supplementary material for: Simulation of nitrogen nuclear spin magnetization of liquid solved nitroxides
Source: Phys Chem Chem Phys. 2021 Jul 29;23(32):17310–22. doi: 10.1039/d0cp06071b (PMC8371994; doi:10.1039/d0cp06071b)
Supplement: CP-023-D0CP06071B-s001 [file CP-023-D0CP06071B-s001.pdf]

Electronic Supplementary Material (ESI) for Physical Chemistry Chemical Physics.
This journal is © the Owner Societies 2021

Supporting Information

Simulation of nitrogen nuclear spin magnetization of liquid solved nitroxides

Andriy Marko, Antonin Sojka, Oleksii Laguta and Petr Neugebauer
Central European Institute of Technology - Brno University of Technology
Purkynova-Str. 23, 61200, Brno, Czech Republic

Contents

S1.	Simulated frequency scan EPR spectra for the electron spin T_2 relaxation time estimation	2
S2.	Excitation and relaxation of nitroxide longitudinal electron spin magnetization	4
S3.	Simulation of experimental data	7
S4.	Comparison of the simulated spectra by direct solution of the LvN equation to the spectra obtained by *EasySpin*	9
S5.	Simulated nitroxide nitrogen NMR lines without electron spin interaction with B_1 field	10
S6.	Determination of the nitrogen nuclear spin T_1 relaxation time	12
S7.	Nitroxide electron saturation factor	13
S8.	Nitroxide nitrogen DNP dependence on B_1 magnitude	14

S1. Simulated frequency scan EPR spectra for the electron spin T_2 relaxation time estimation

This section contains simulated frequency scan CW-EPR spectra, which were used to illustrate our computational method and to estimate electron spin T_2 relaxation times according to the formula (30) of the main text.

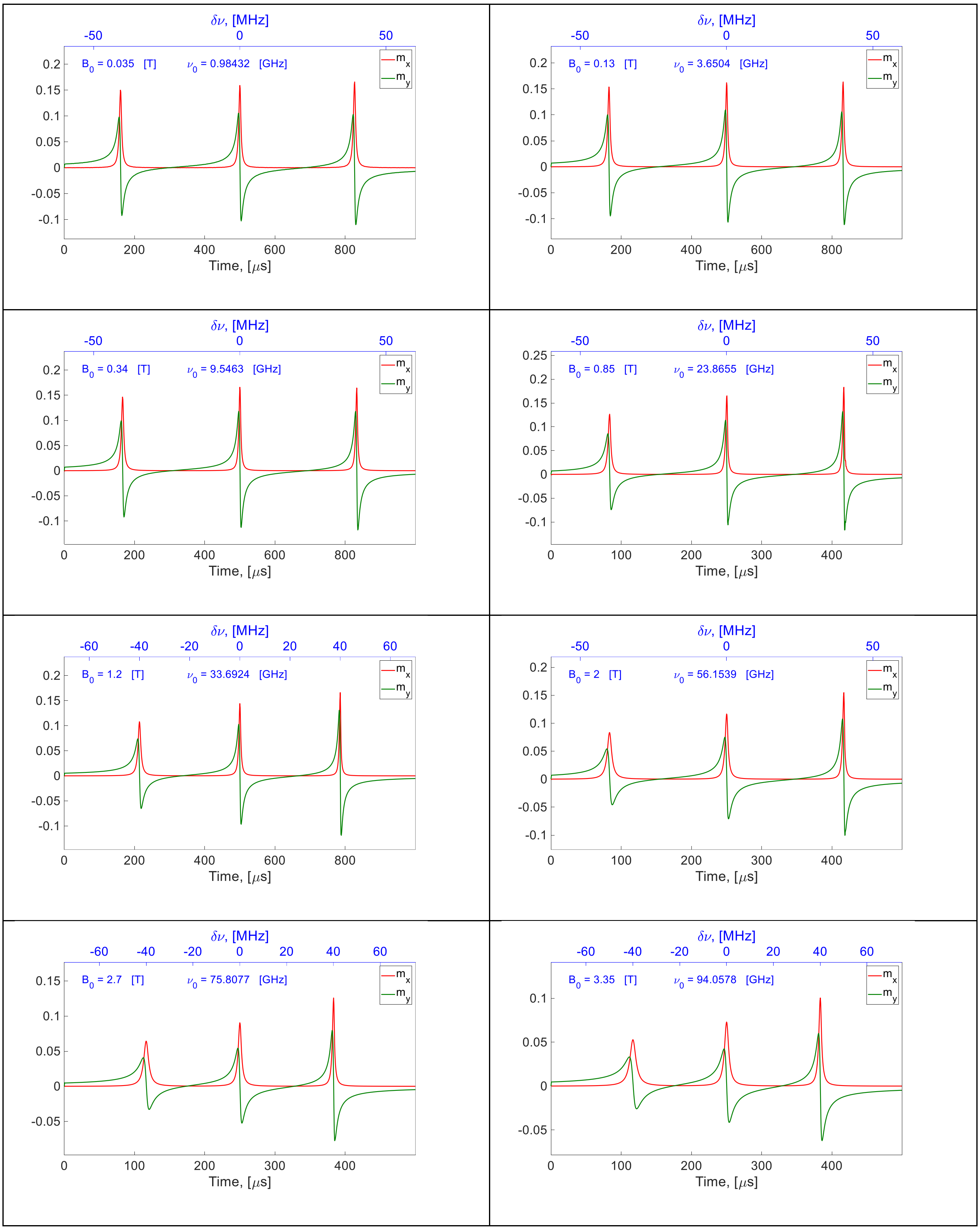

See the next page …

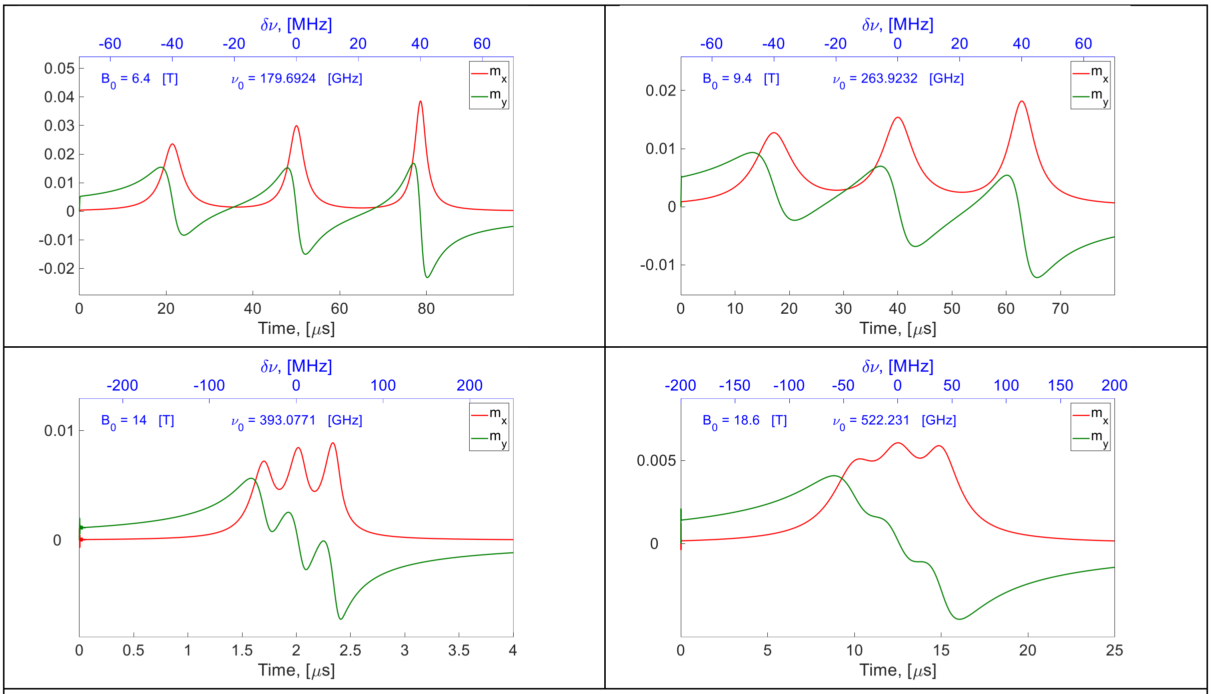

Figure S1. Simulated evolution of the transverse electron spin magnetization of liquid solved nitroxides in CW-EPR experiments with varied MW frequency. The values of B_0, ν_0, $\dot{\nu}$ and $\Delta\nu$ can be deduced from the data in the plots. $B_1 = 2 \times 10^{-5}$ T and $\Phi_0 = \frac{\pi}{2}$ were used in all simulations. The relaxation superoperator $\hat{\hat{\hat{\mathrm{R}}}}$ was determined for each B_0 with K = 27000, T = 150 ps, $\Delta\tau = 0.1$ ps, $\sigma_\phi = 2.3°$, $\mathrm{I} \approx 4.975 \times 10^{-45}$ kg*m^2, $\tau_c^\eta = 30$ fs, $\mathcal{T}$ = 300 K in all cases.

S2. Excitation and relaxation of nitroxide longitudinal electron spin magnetization

The left column of this table contains the simulation of the longitudinal nitroxide electron spin magnetization $M_z(t)$ upon application of t_p = 5 μs (2.5 μs for $B_0 \geq 2$ T) long MW pulse followed by an observation period when the irradiation is switched off. The right column shows the logarithm of the function $Y(t) = \left(M_{eq} - M_z(t)\right)/\left(M_{eq} - M_z(t_p)\right)$ for the time $t \geq t_p$.

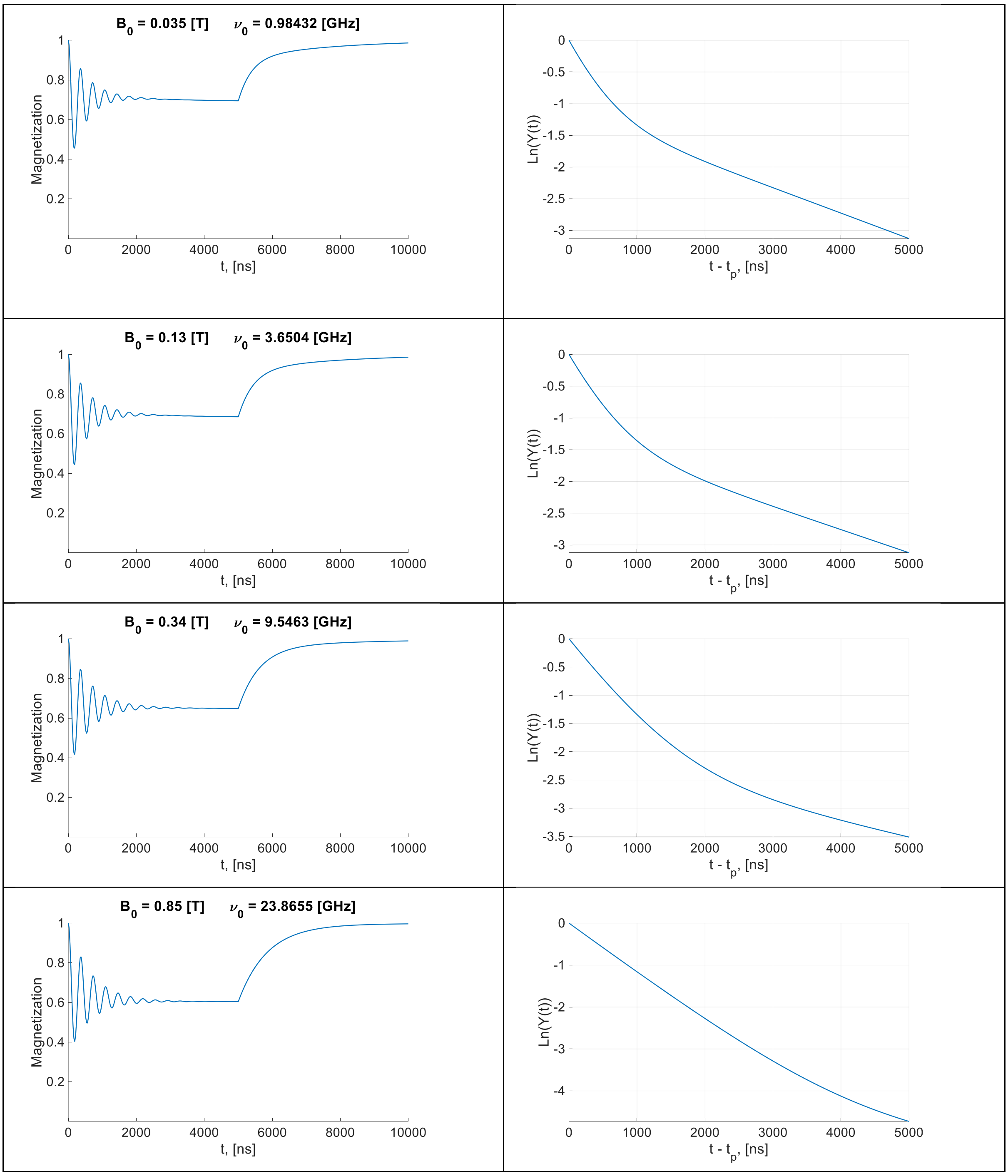

See the next page …

See the previous page …

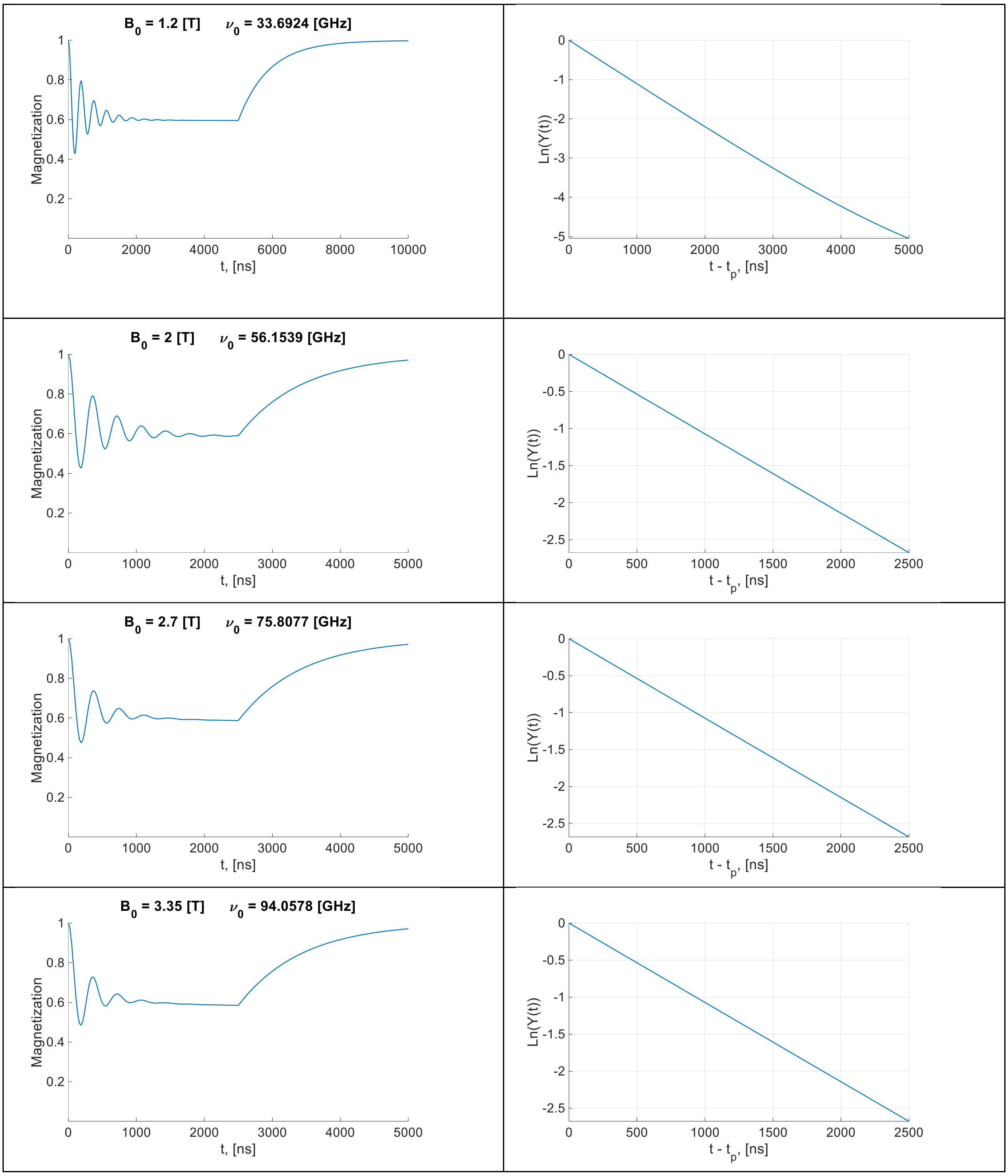

See the next page …

See the previous page …

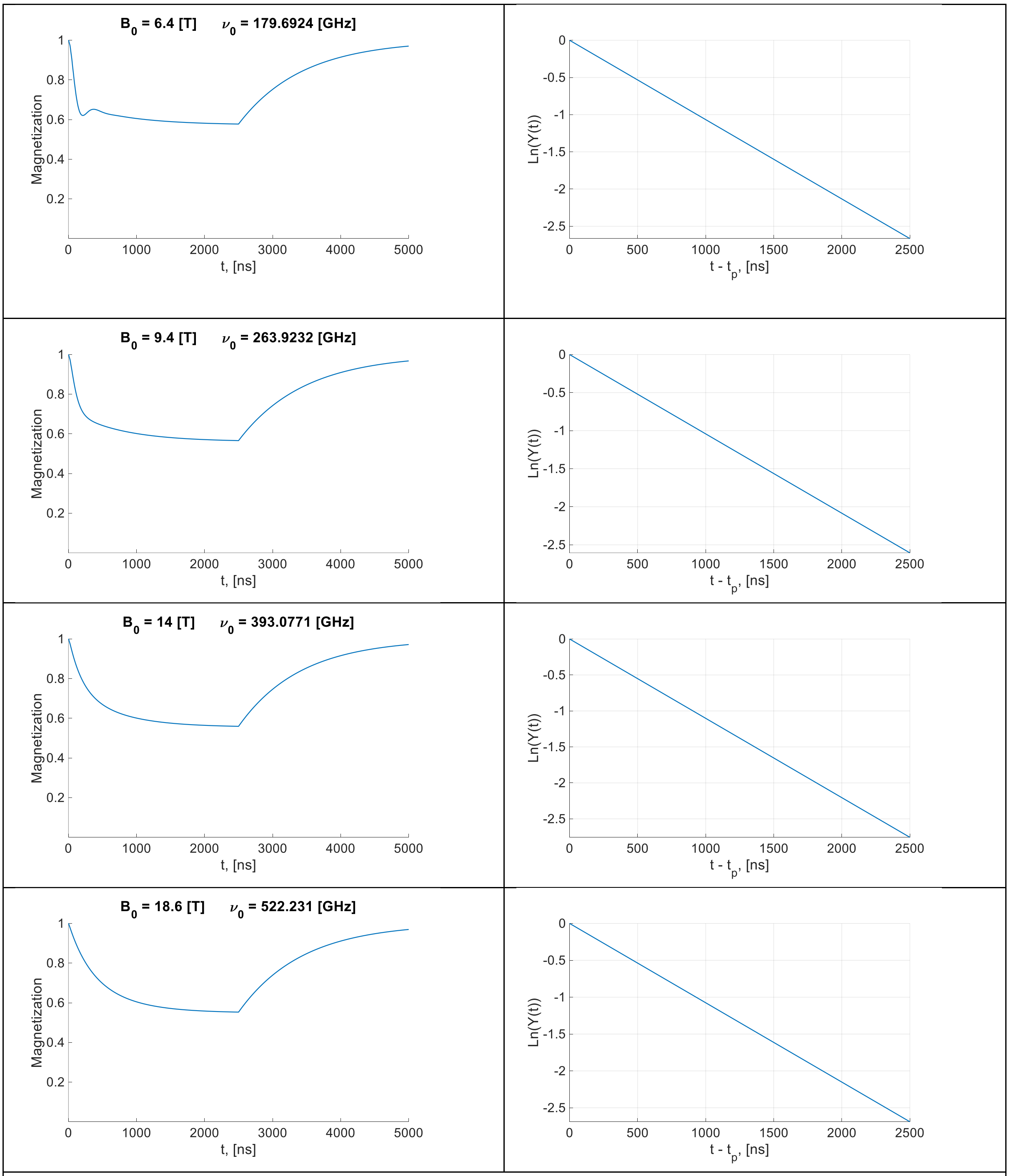

Figure S2. Simulated recovery of the longitudinal electron spin magnetization upon the application of a linearly polarized MW field with B_1 = 0.0002 T tuned to the central nitroxide line frequency ν_0, which is determined for each B_0 value and indicated in the plots. The computations were performed with the same relaxation superoperators $\widehat{\widehat{\mathrm{R}}}$ as the corresponding spectra in the Figure S1.

S3. Simulation of experimental data

This section demonstrates the simulation of the real experimental data using the computational method based on the direct solution of the LvN equation developed in this paper.

a) TEMPO in water in B_0 = 0.337 T

The experimental data were obtained by measuring X-band CW-EPR spectra of water solved TEMPO with a concentration of 1mM at 290 K. For the simulation of these experimental data, we used the simulation parameters given in the table

Magnetic field, B_0	0.337 T
Linearly polarized magnetic field, B_1	4 microT
g-tensor, diag(g)	[2.0088 2.006 2.002]
Hyperfine interaction tensor, diag(A)/(2π)	[17.9 17.9 107.2] MHz
^{14}N Quadrupole interaction tensor, diag(Q)/(2π)	[0.1 1.6 -1.7] MHz
Temperature, T	290 K
Random trajectory time increment, Δτ	0.1 ps
Random trajectory angle change, σ_ϕ	2.1° (corresponds to τ_c = 24.8 ps)
Angular velocity correlation time, τ_c^η	35 fs

With these parameters we simulated the evolution of the transverse electron spin magnetization in the frequency scan CW-EPR experiment shown in Fig.3A (left). The frequency sweep time and range are indicated at the figure bottom and top axes, respectively. The curves m_x(δν) and m_y(δν) were transformed into the field sweep spectra m_x(δB) and m_y(δB) by flipping the x-axis and rescaling MHz to Gauss. Further, the derivative spectra were computed and an additional Gaussian line broadening of 0.08 mT (equal for all three lines) was introduced by convoluting the derivative spectra with a Gaussian curve. The normalized result of this computation compared to the experimental spectrum is shown in Fig. 3A (right).

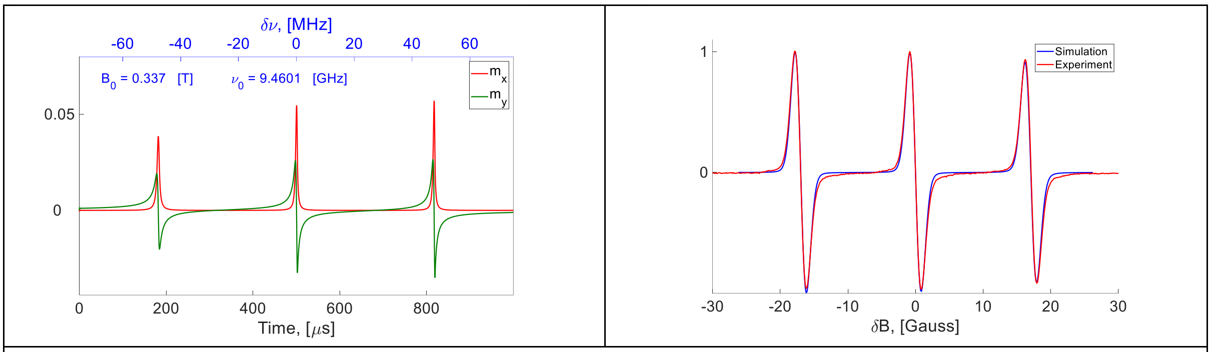

Figure S3.A (left) Simulated evolution of the transverse electron spin magnetization with the parameters specified in the table above. (right) The derivative spectrum (obtained from m_x(δν) shown left as described in the text above) compared to the experimental data.

b) TEMPOL in water in B_0 = 9.2 T

Here we simulated the field scan CW EPR spectrum of low concentrated water solved TEMPOL at B_0 = 9.2 T and room temperature published by [c1]*. The simulation parameters are listed in the table below

Magnetic field, B_0	9.2 T
Linearly polarized magnetic field, B_1	20 microT
g-tensor, diag(*g*)	[2.0088, 2.006, 2 .002]
Hyperfine interaction tensor, diag(*A*)/(2π)	[17.7, 17.7, 105.1] MHz
^{14}N Quadrupole interaction tensor, diag(*Q*)/(2π)	[0.1 1.6 -1.7] MHz
Temperature, $\mathcal{T}$	300 K
Random trajectory time increment, $\Delta\tau$	0.1 ps
Random trajectory angle change, σ_ϕ	2.3° (corresponds to $\tau_c = 20$ ps)
Angular velocity correlation time, τ_c^η	30 fs

In our simulation we used the same values for *g*-tensor and hyperfine tensor *A* as were used in []. The relaxation superoperator $\hat{\hat{R}}$ was determined for the magnetic field B_0 = 9.2 T with the same parameters *K*, *T*, $\Delta\tau$, σ_ϕ, I, τ_c^η and $\mathcal{T}$, which are specified in the caption of the Fig. S1. Note that these parameters ($\Delta\tau = 0.1$ ps and $\sigma_\phi = 2.3°$) yield a rotational correlation time $\tau_c = 20$ ps, is the same as used in [c1]. With these parameters the frequency scan absorption and dispersion EPR spectra were simulated (see Fig. S3.B, left). Similar as in the previous case, these spectra were transformed into the field sweep spectra. To account for the admixture of the dispersion component in the measured data, the function $S = m_x\cos(\alpha) + m_y\sin(\alpha)$, where α is the phase correction angle, was computed. In order to introduced additional broadening, the spectrum S was convoluted with a Lorenz curve with a width of 0.08 mT, which is the same value as used in [c1]. Further the derivative of the spectrum was computed, normalized by its maximal value and compared to the experimental data (see Fig S3.B, right).

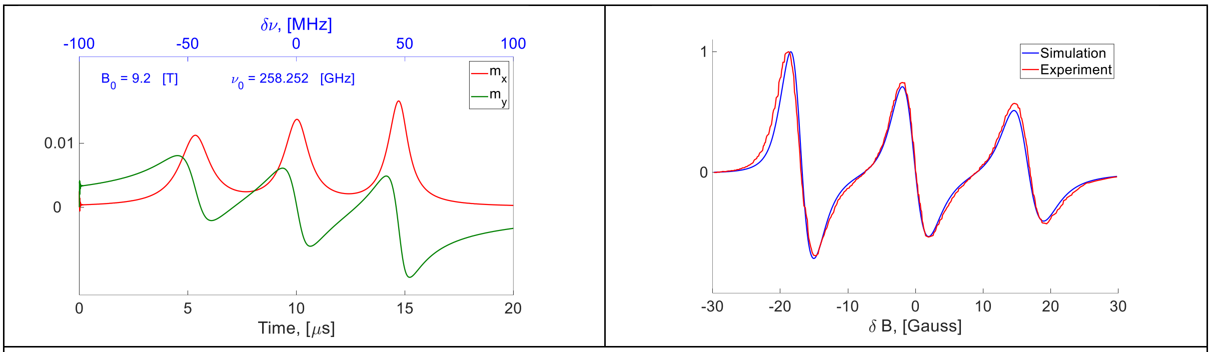

Figure S3.B (left) Simulated evolution of the transverse electron spin magnetization of liquid solved TEMPOL in the frequency scan CW-EPR experiment at B_0 = 9.2 T with the parameters specified in the text above. (right) The experimental spectrum (red) and the computed spectrum (blue) obtained from the curves shown left by building a superposition $m_x\cos(\alpha) + m_y\sin(\alpha)$ with α =15°, computing derivatives and introducing line broadening as described in the text.

*[c1] = D. Sezer et al, PCCP 2009, Vol. 11, p. 6638

S4. Comparison of the simulated spectra by direct solution of the LvN equation to the spectra obtained by EasySpin

In this section we compared our simulated nitroxide CW-EPR spectra, which we obtained by the direct solution of the LvN equation, to the corresponding spectra obtained by EasySpin package. Four CW-EPR spectra for B_0 = 1.2 T, B_0 = 3.35T, B_0 = 9.4 T and B_0 = 14 T, which are shown in Fig S1, were also simulated with the EasySpin package ('garlic') using the same *g*- tensor, hyperfine interaction tensor *A* and rotational correlation time as we used for the simulation of the Fig. S1 spectra. Additionally, we introduced 0.02 mT Lorentzian line broadening for all spectra in EasySpin simulation, which did not account for the effect of the B1 field value on the spectral shape. To remind you, our spectra in Fig. S1 were simulated with B1 = 0.02 mT.

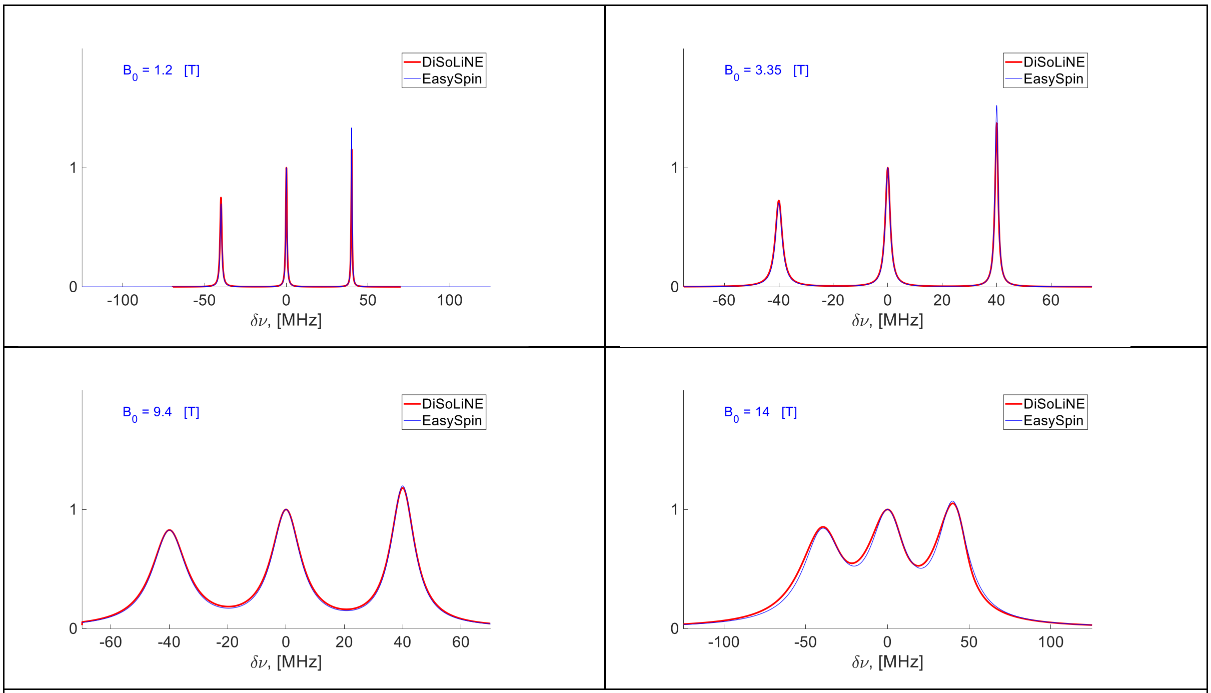

Figure S4. Red curves with DiSoLiNE legend are the same as in Fig S1. Blue curves are the spectra obtained by the EasySpin package with the same g-tensor, hyperfine interaction tensor and rotational correlation time.

S5. Nitroxide nitrogen NMR lines simulated with and without electron spin interaction with B_1 field

This chapter illustrates the frequency scan CW NMR spectra, which were simulated with (left) and without (right) electron spin interaction with the field B_1(t), that is, with the Hamiltonians $\hat{H}_{irr}(t) = -\mathbf{B}_1(t)(\gamma_N\hat{\mathbf{I}} + \gamma_e\hat{\mathbf{S}})$ and $\hat{H}_{irr}(t) = -\mathbf{B}_1(t)\gamma_N\hat{\mathbf{I}}$, respectively.

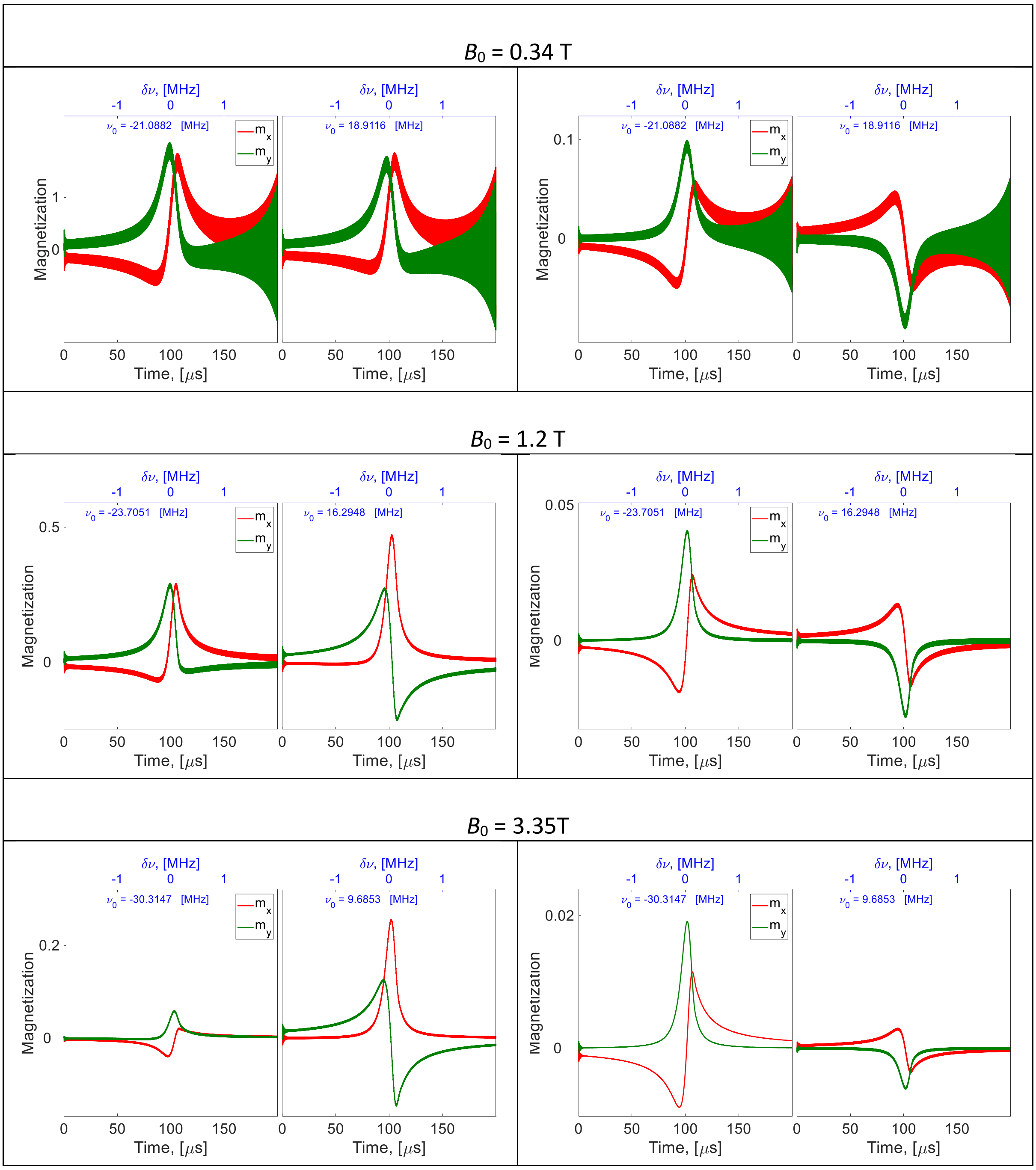

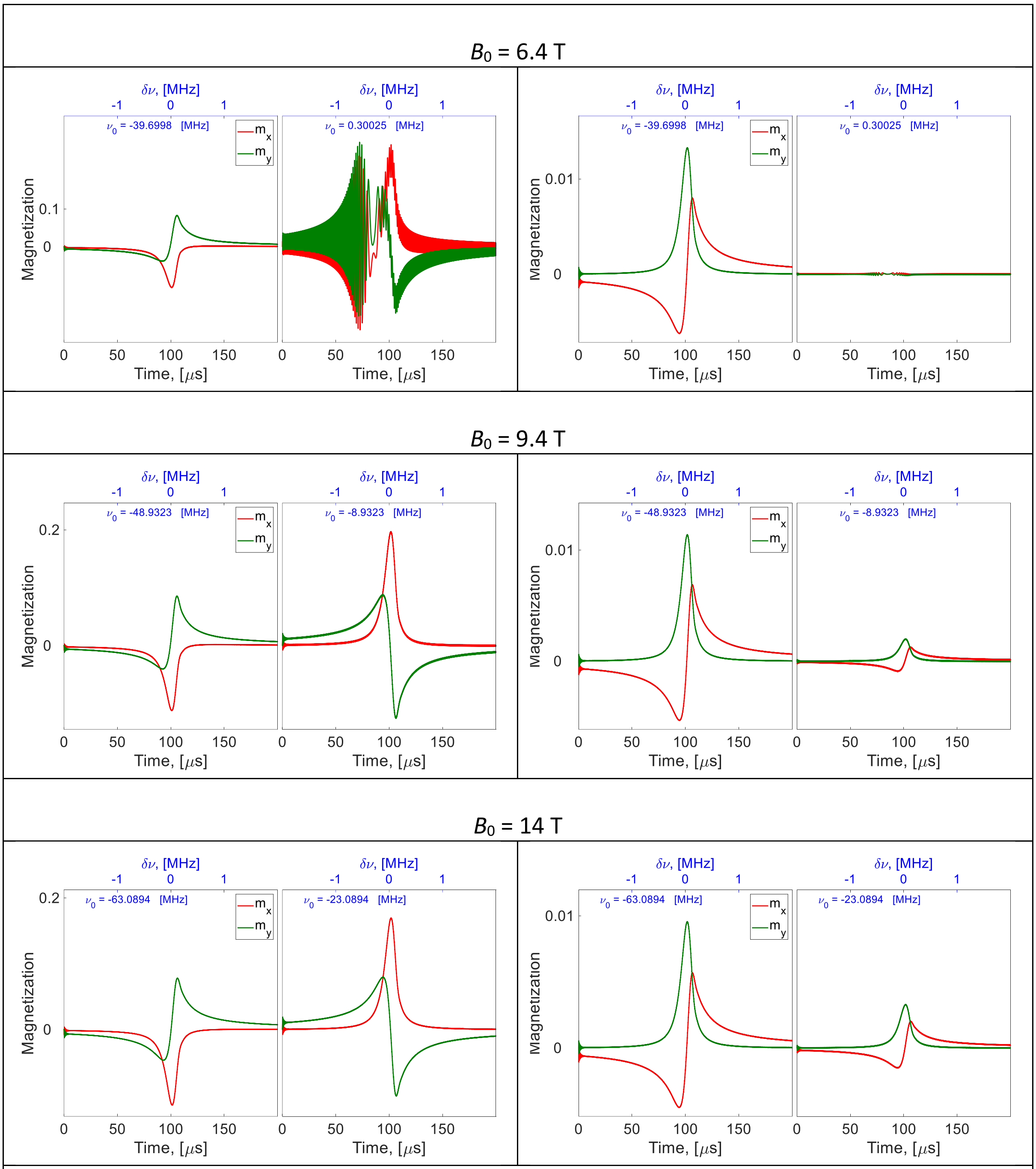

Figure S5. Simulated transverse nitroxide nitrogen nuclear spin magnetization (m_x – red, m_y – green) upon application of a linearly polarized RF field with B_1 = 0.001 T, Φ_0 = 0. The central frequency ν_0, the frequency scan range $\Delta\nu$ and rate $\dot{\nu}$ can be read out (derived) from each figure data. The computations were performed with the same relaxation superoperators $\hat{\hat{\mathrm{R}}}$ as the corresponding spectra in the Figure S1 and S2.

S6. Determination of the nitrogen nuclear spin T_1 relaxation time

The plots on the left side illustrate the time dependence of the simulated longitudinal nitrogen nuclear spin magnetization, M_z^N , during 25 μs long MW irradiation time (t_p = 25 μs) followed by a 25 μs long period when the irradiation is switched off and the nitroxide spin system returns to the thermal equilibrium. The plots on the right side show the corresponding logarithm of the function $Y(t) = \left(M_{eq} - M_z^N(t)\right)/\left(M_{eq} - M_z^N(t_p)\right)$ for the time $t \geq t_p$, which allows us to determine nuclear T_1 relaxation time as the point where the function Ln(Y(t)) is equal to -1. In this way, we obtained T_1 = 6.7 μs for B_0 = 1.2 T and T_1 = 7.0 μs for B_0 = 14 T.

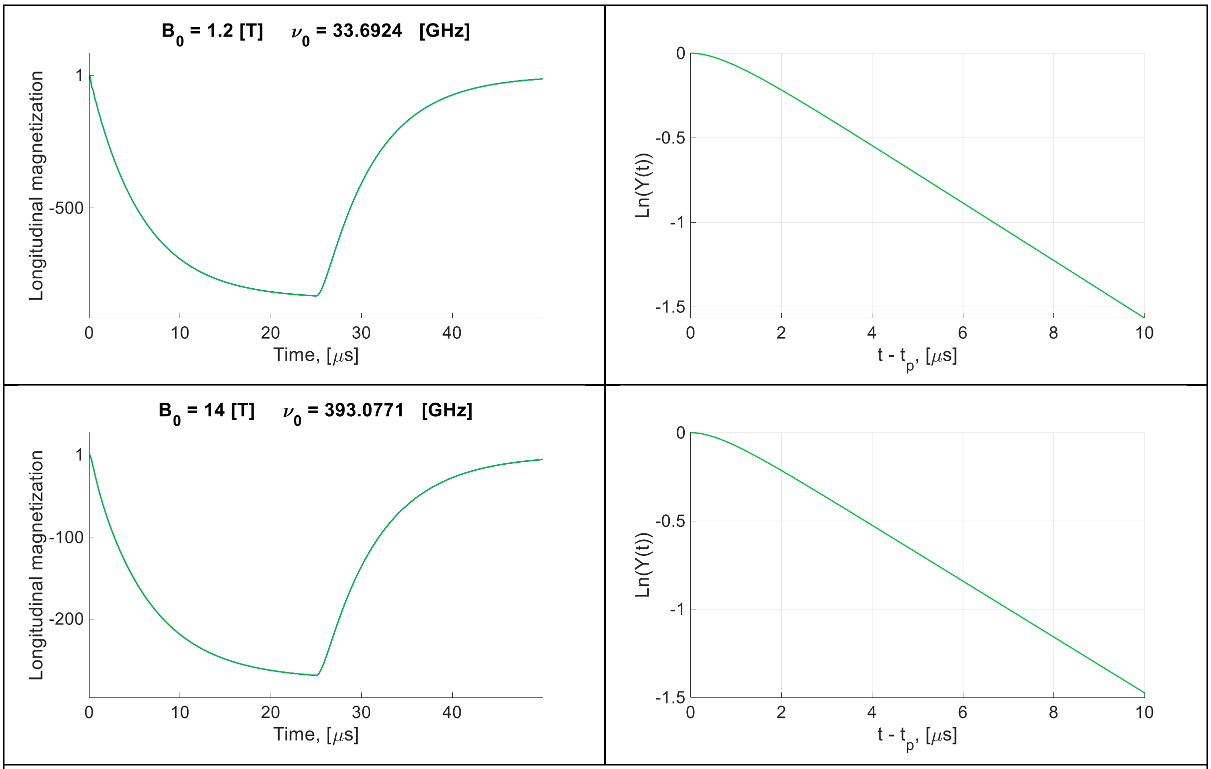

Figure S6. (left) Time dependence of the longitudinal nitroxide nitrogen nuclear spin magnetization upon the MW, which is applied the central nitroxide EPR line for 25 μs and then switched off. The simulation of these curves was performed with the same parameters as the corresponding blue curves shown in the main text. (right) Auxiliary function Ln(Y(t)), which is plotted to determine nitrogen nuclear spin relaxation time T_1.

S7. Nitroxide electron spin saturation factor

Simulated longitudinal nitroxide electron spin magnetization, which was used to determine the saturation factors shown in the TABLE I. In this simulation, the nitroxide central EPR line is permanently irradiated with the microwave.

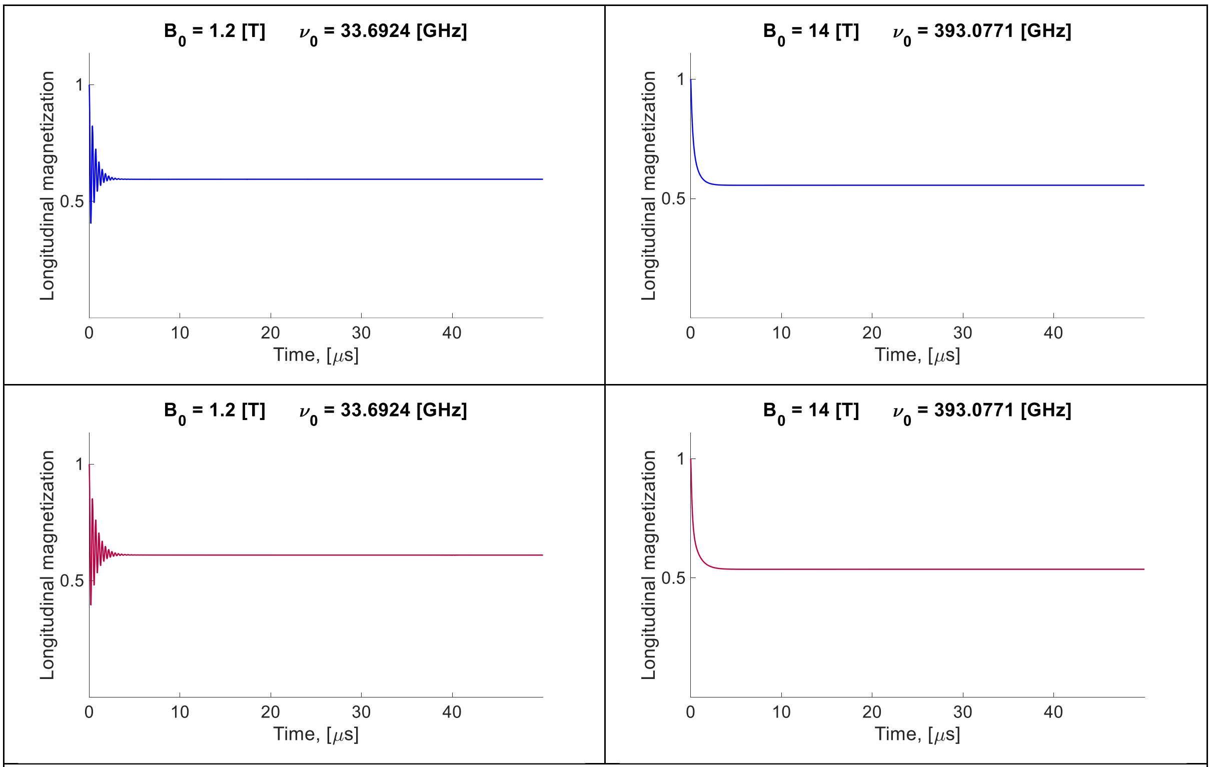

Figure S7. Time dependence of the longitudinal electron spin magnetization under the action of the MW irradiation with B_1 = 0.0002 T. The upper two plots were simulated with the same relaxation superoperators $\hat{\hat{R}}$ as the corresponding spectra in the Figure S1, S2, S5, and S6. The lower two plots were simulated with the relaxation superoperators $\hat{\hat{R}}$, determined by a faster rotational diffusion, specified in the caption of FIG. 8 of the main text.

S8. Nitroxide nitrogen DNP dependence on B_1 magnitude

This section illustrates the time dependence of the longitudinal nitrogen spin magnetization in the presence of MW electron spin irradiation with a stronger and a weaker B_1 magnitude. The magnetization is normalized to its initial thermal equilibrium value.

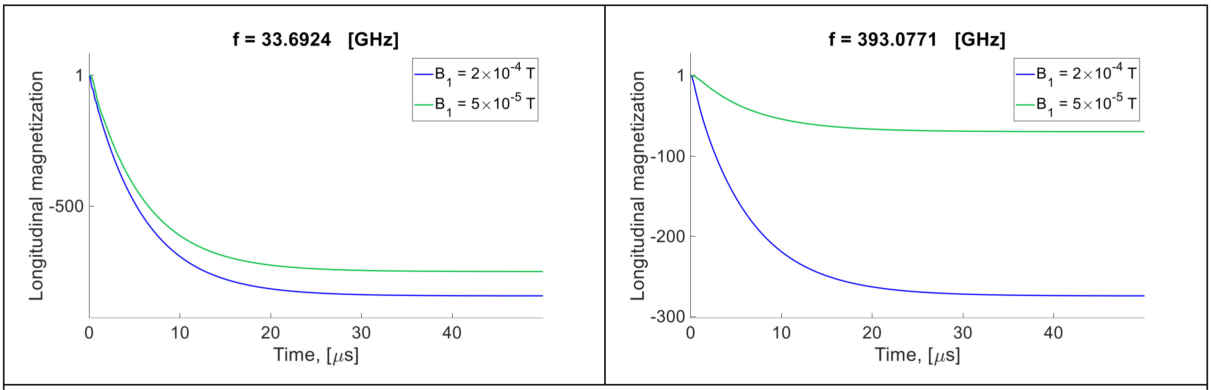

Figure S8. Time dependence of the longitudinal nitroxide nitrogen nuclear spin magnetization upon the MW, which is used to irradiate the central nitroxide EPR line. The blue curves in both figures are equal to the blue curves in the FIG. 9 of the main text. The green curves were computed with the same relaxation superoperators $\hat{\hat{R}}$ as the blue curves.
